# Metatranscriptomics-guided discovery and characterization of a polyphenol-metabolizing gut microbial enzyme

**DOI:** 10.1016/j.chom.2024.10.002

**Published:** 2024-10-28

**Authors:** Minwoo Bae, Chi (Chip) Le, Raaj S. Mehta, Xueyang Dong, Lindsey M. Pieper, Lorenzo Ramirez, Margaret Alexander, Sina Kiamehr, Peter J. Turnbaugh, Curtis Huttenhower, Andrew T. Chan, Emily P. Balskus

**Affiliations:** 1 Department of Chemistry and Chemical Biology, Harvard University, Cambridge, MA 02138, USA; 2 Broad Institute of MIT and Harvard, Cambridge, MA 02142, USA; 3 Division of Gastroenterology, Massachusetts General Hospital and Harvard Medical School, Boston, MA 02114, USA; 4 Clinical and Translational Epidemiology Unit, Massachusetts General Hospital, Boston, MA 02114, USA; 5 Department of Microbiology & Immunology, University of California San Francisco, San Francisco, CA 94143, USA; 6 Chan Zuckerberg Biohub-San Francisco, San Francisco, CA 94158, USA; 7 Department of Biostatistics, Harvard T.H. Chan School of Public Health, Boston, MA 02115, USA; 8 Department of Immunology and Infectious Diseases, Harvard T.H. Chan School of Public Health, Boston, MA 02115, USA; 9 Harvard Chan Microbiome in Public Health Center, Harvard T.H. Chan School of Public Health, Harvard University, Boston, MA 02115, USA; 10 Channing Division of Network Medicine, Department of Medicine, Brigham and Women’s Hospital and Harvard Medical School, Boston, MA 02115, USA; 11 Howard Hughes Medical Institute, Harvard University, Cambridge, MA 02138, USA; 12 These authors contributed equally; 13 Lead contact

## Abstract

Gut microbial catechol dehydroxylases are a largely uncharacterized family of metalloenzymes that potentially impact human health by metabolizing dietary polyphenols. Here, we use metatranscriptomics (MTX) to identify highly transcribed catechol dehydroxylase-encoding genes in human gut microbiomes. We discover a prevalent, previously uncharacterized catechol dehydroxylase (*Gp* Hcdh) from *Gordonibacter pamelaeae* that dehydroxylates hydrocaffeic acid (HCA), an anti-inflammatory gut microbial metabolite derived from plant-based foods. Further analyses suggest the activity of *Gp* Hcdh may reduce anti-inflammatory benefits of polyphenol-rich foods. Together, these results show the utility of combining MTX analysis and biochemical characterization for gut microbial enzyme discovery and reveal a potential link between host inflammation and a specific polyphenol-metabolizing gut microbial enzyme.

## eTOC

Bae et al. uncover that a highly transcribed member (*Gp* Hcdh) of the gut bacterial catechol dehydroxylase family metabolizes hydrocaffeic acid, an abundant anti-inflammatory polyphenol. Analyses of human data suggest that the activity of *Gp* Hcdh may reduce anti-inflammatory activity of foods that are major sources of hydrocaffeic acid.

## Graphical Abstract

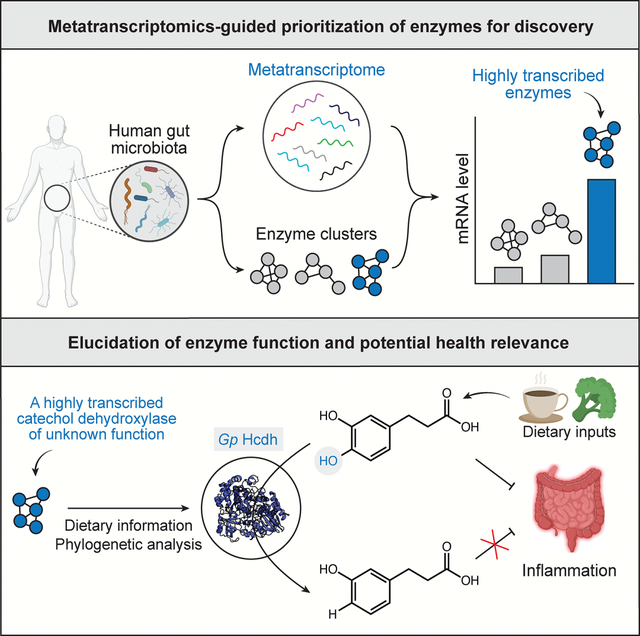

## Introduction

**Figure 1.**
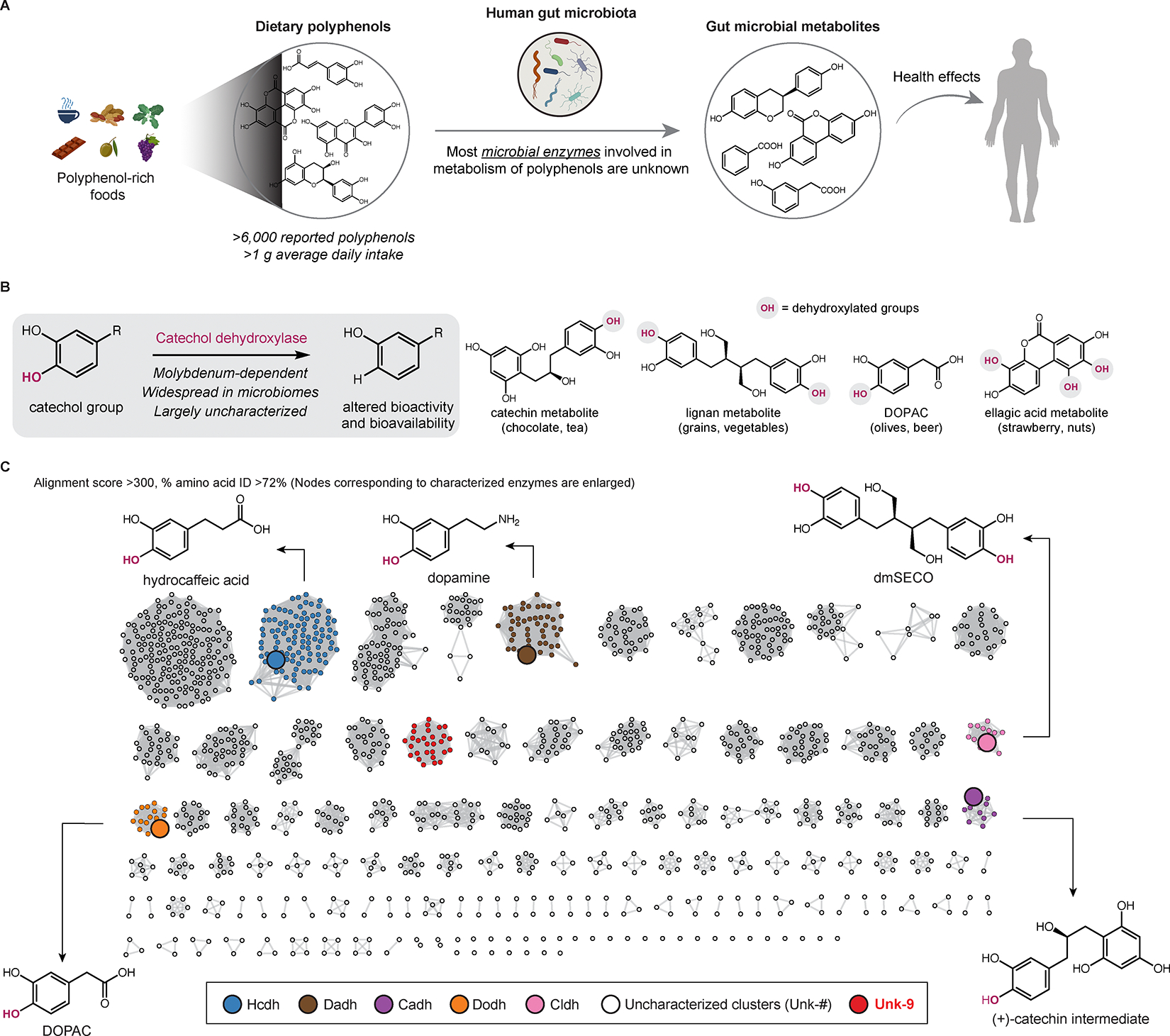
Most gut bacterial catechol dehydroxylases are uncharacterized. (A) The human gut microbiota metabolizes polyphenols. (B) The general transformation catalyzed by catechol dehydroxylases and example polyphenol substrates. (C) SSN of catechol dehydroxylases found in the human gut. See also Figure S1 and Table S1.

Polyphenols are a diverse group of metabolites (>6,000 reported compounds) characterized by phenol functional groups (Figure 1A). They are abundant in plant-based foods such as vegetables, coffee, and tea^1^, with an average daily intake >1 gram^2,3^. Dietary polyphenols have been associated with improved health outcomes, including reduced inflammation, lower risk of cardiovascular diseases, and healthy aging^4,5^. Importantly, the biological impacts of polyphenols may depend on gut microbial metabolism due to their poor oral bioavailability and extensive modification by the gut microbiota^6,7^. Indeed, the gut microbiota has been associated with inter-individual variation in the health effects of polyphenols^8,9^. However, the gut microbial enzymes involved in polyphenol metabolism are largely unknown^10^.

A central reaction in gut microbial polyphenol metabolism is catechol dehydroxylation, in which the *para*-hydroxyl group of a catechol (1,2-dihydroxylated aromatic ring) is replaced by a hydrogen atom (Figure 1B). Many catechol-containing polyphenols undergo dehydroxylation in the human body^11^, a process first linked to the gut microbiota in antibiotic-treated rats^12^. Catechol dehydroxylation often drastically alters the bioactivity and bioavailability of polyphenol metabolites^11^. Furthermore, this activity is highly variable between humans^13–15^, which may contribute to interindividual variation in responses to polyphenol consumption. Recent efforts linked catechol dehydroxylation to the molybdenum-dependent catechol dehydroxylase enzyme family and showed individual enzymes are upregulated in response to their specific catechol substrates^13,14,16^. Despite this progress, only a few catechol dehydroxylases have been biochemically characterized^16^.

Here, we employ sequence similarity network (SSN) analysis and MTX analysis of human gut microbiome datasets to prioritize highly transcribed, widely distributed catechol dehydroxylases for characterization. We identify a highly transcribed and prevalent catechol dehydroxylase (*Gp* Hcdh) and determine its substrate as the common dietary polyphenol metabolite HCA. By analyzing a human gut microbiome dataset, we uncover a potential link between this enzyme and a reduction in the anti-inflammatory benefits of consuming polyphenol-rich vegetables. This integrated computational and experimental approach emphasizes the potential of integrating MTX with biochemical studies for discovery of important gut microbial enzymes. This work further demonstrates how knowledge of gut microbial enzyme function can inform mechanistic hypotheses underlying gut microbiota-host interactions.

## Results

### Most gut bacterial catechol dehydroxylases are uncharacterized

We initially sought to understand the diversity of catechol dehydroxylases in the human gut microbiome. Catechol dehydroxylases are members of the dimethyl sulfoxide (DMSO) reductase superfamily and are predicted to harbor a bis-molybdopterin guanine dinucleotide (bis-MGD) cofactor^13,16,17^. Following the initial discovery of dopamine dehydroxylase (Dadh) in the gut Coriobacteriia *Eggerthella lenta* in 2019^16^, four additional catechol dehydroxylases have been linked to the dehydroxylation of specific dietary polyphenols in *E. lenta* (HCA dehydroxylase (Hcdh) and (+)-catechin dehydroxylase (Cadh)) and another Coriobacteriia *Gordonibacter pamelaeae* (DOPAC dehydroxylase (Dodh) and catechol lignan dehydroxylase (Cldh))^13,14^. However, most putative catechol dehydroxylases in *Gordonibacter* and *Eggerthella* genomes remain uncharacterized^13^.

To compile a comprehensive list of gut microbial catechol dehydroxylase homologs, we searched a dataset of >150,000 human-associated metagenome-assembled genomes (MAGs) using protein sequences of the five characterized catechol dehydroxylases as queries^18^. This search yielded 2,903 unique sequences from Coriobacteriia MAGs (Figure S1A). Multiple homologs were from *G. pamelaeae* and *E. lenta* as well as other Coriobacteriia known to dehydroxylate catechols in culture, such as *Adlercreutzia equolifaciens* and *Ellagibacter isourolithinifaciens*^19–21^. Notably, homologs were also found in species not yet reported to perform this metabolism (Figure S1A).

Next, we sought to classify catechol dehydroxylase homologs using sequence similarity network (SSN) analysis^22^. The minimum alignment score and amino acid (aa) sequence identity were optimized to build an SSN in which catechol dehydroxylases in the same cluster are predicted to share the same biochemical function (Figure S1B and Figure S1C). With a minimum alignment score and aa sequence identity of 300 and 72%, respectively, we separated the five characterized catechol dehydroxylases, which have different substrates, into different clusters (Figure 1C). The resulting SSN contained 126 distinct enzyme clusters.

To investigate whether SSN clusters contain isofunctional enzymes, we examined the dehydroxylating activities of bacterial isolates with sequences from clusters containing characterized enzymes. For example, in addition to sequences from known metabolizers, the Hcdh, Dadh, and Dodh clusters include sequences from cultured bacteria that have not been reported to dehydroxylate their corresponding substrates (Figure S1D). These bacterial cultures dehydroxylated the predicted substrates, suggesting these enzyme clusters are likely isofunctional (Figure S1E). Although we cannot verify all clusters, our SSN broadly reflects the functional diversity of gut microbial catechol dehydroxylases. Notably, our SSN analysis revealed that most catechol dehydroxylase clusters (n=121/126) are of unknown function (Figure 1C and Figure S1F), underscoring the need to prioritize enzymes for discovery.

### Metatranscriptomics analysis reveals an uncharacterized catechol dehydroxylase that is highly expressed and prevalent in the human gut

**Figure 2.**
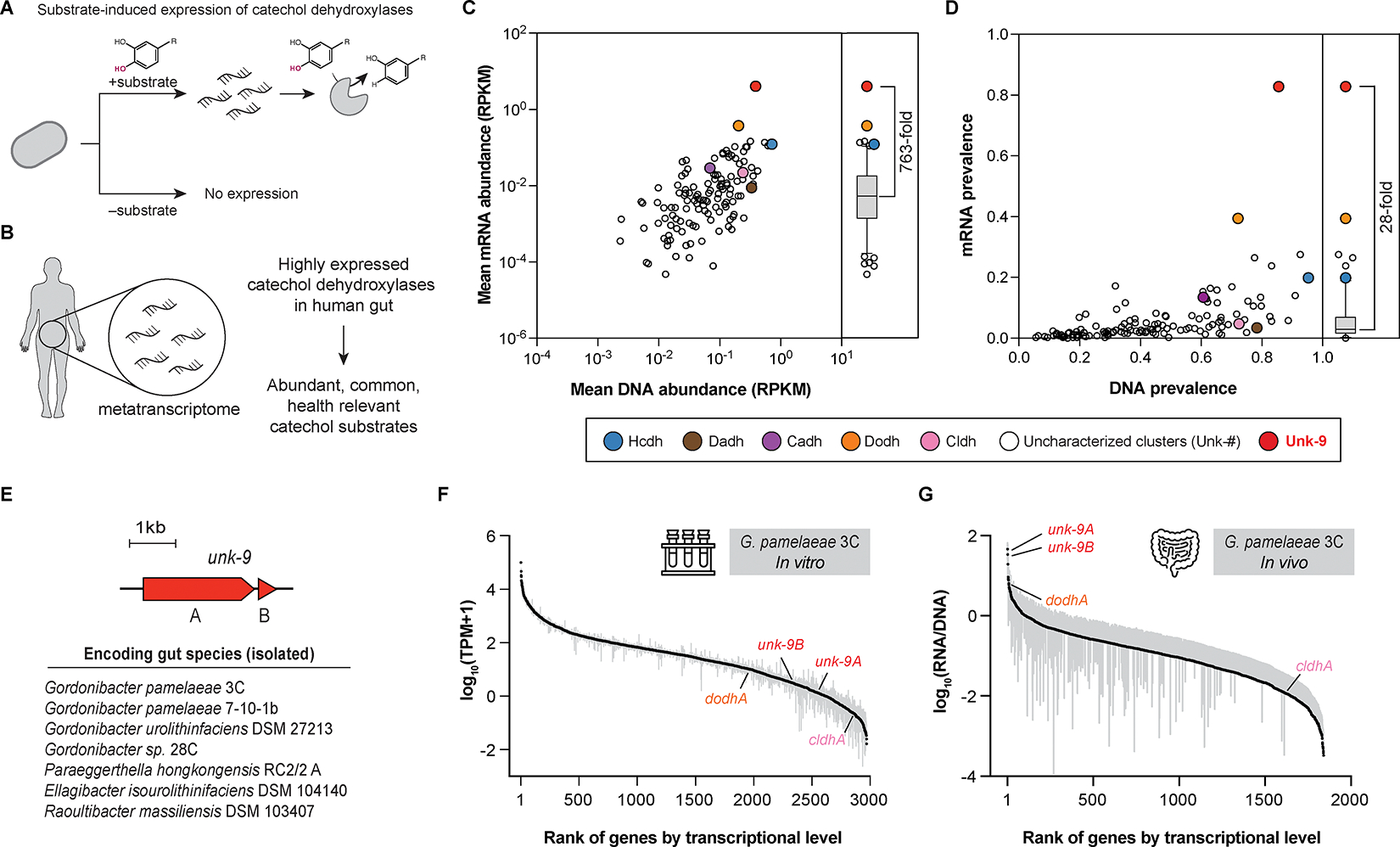
MTX analysis reveals an uncharacterized catechol dehydroxylase that is highly expressed and prevalent in the human gut. (A) The expression of catechol dehydroxylases is induced by their substrates. (B) MTX-based prioritization of an uncharacterized catechol dehydroxylase. (C–D) Analysis of DNA and mRNA abundance (C) and prevalence (D) of catechol dehydroxylase clusters using the MLVS dataset. Bars represent median. RPKM, reads per kilobase million. (E) List of Unk-9 encoding gut species. (F–G) Ranked median transcriptional levels of the whole protein-coding genes of *G. pamelaeae* 3C *in vitro* (F) and *in vivo* (G), grey bar represents 95% confidence interval. TPM, transcripts per kilobase million. See also Figure S2 and Table S2.

Recognizing that the expression of individual catechol dehydroxylase genes is highly induced by their substrates in bacterial culture (Figure 2A)^13,14,16,23^, we applied an MTX-based prioritization strategy^24,25^. We hypothesized that prevalent, highly transcribed catechol dehydroxylases in human gut MTX datasets likely metabolize common, abundant dietary polyphenols as prior studies have shown that *in vivo* MTX reflects substrate availability for some gut microbial enzymes (Figure 2B)^24–27^. However, this has not yet been demonstrated for catechol dehydroxylases.

To assess DNA and mRNA levels of catechol dehydroxylase clusters from our SSN in human gut microbiomes, we used metagenomics (MGX) and MTX data from the Men’s Lifestyle Validation Study (MLVS), a cohort of 307 healthy men enrolled in the Health Professionals Follow-Up Study^28^. Cluster-level quantification showed DNA and mRNA abundances of the clusters were generally correlated (Pearson r = 0.58), consistent with previous findings^29,30^. Notably, a few outliers, such as the Dodh cluster, showed higher mRNA levels compared to clusters with similar DNA levels (Figure 2C). Dodh transcripts were detected in 40% of the MTX samples, whereas the expression of most clusters (120/126) was detected in <20% of samples (Figure 2D). The substrate of Dodh, 3,4-dihydroxyphenylacetic acid (DOPAC), is a major gut microbial metabolite of quercetin and dopamine and was detected in all urine samples from a previous clinical study^31,32^. This supports our hypothesis that prominent catechol dehydroxylases can be discovered using MTX-based prioritization.

Our analysis showed certain catechol dehydroxylase clusters containing uncharacterized enzymes were more highly and widely transcribed in the human gut than other clusters. Notably, cluster Unk-9 had the highest mRNA abundance (763-fold higher than the median) and mRNA prevalence (detected in 82.8% of the MTX samples) among all clusters (Figure 2C, Figure 2D, and Figure S2A). Unk-9 also had the highest mRNA/DNA ratio (242-fold higher than the median) (Figure S2B). This trend was confirmed in another dataset from the Human Microbiome Project 2 (HMP2)^33^, where Unk-9 was the most transcribed catechol dehydroxylase cluster (Figure S2C). Unk-9 did not have the highest DNA abundance and prevalence, emphasizing the importance of MTX in highlighting this enzyme.

Predicted to comprise a catalytic subunit (Unk-9A) that binds the bis-MGD cofactor and an accessory subunit (Unk-9B), Unk-9 is encoded by several cultured gut Coriobacteriia including *G. pamelaeae* 3C and other *Gordonibacter* strains, *Paraeggerthella hongkongensis* RC2/2 A, *E. isourolithinifaciens* DSM 104140, and *Raoultibacter massiliensis* DSM 103407 (Figure 2E). Data from our previous MAG search indicated that Unk-9 sequences are globally distributed, found in gut microbiomes from North America, Europe, Asia, as well as from agro-pastoral Mongolian and Malagasy cohorts (Figure S2D).

Knowing that Unk-9 is the most highly expressed catechol dehydroxylase *in vivo*, we examined its expression in bacterial culture to determine if its substrate is present in culture media. We analyzed the transcriptional levels of all protein-coding genes in the *G. pamelaeae* 3C strain grown in a modified Brain–Heart Infusion medium (*in vitro*)^14^. Interestingly, genes encoding Unk-9A and Unk-9B were among the least expressed ones *in vitro* (2576^th^ and 2316^th^ out of 2979 genes, respectively) (Figure 2F). In contrast, these genes were the top two most expressed *G. pamelaeae* 3C genes in the MLVS data (*in vivo*)^28^ (Figure 2G), suggesting that Unk-9 expression is induced by an external stimulus present in the gut but not in the culture medium. Altogether, its high *in vivo* expression and global distribution led us to investigate the biochemical function of Unk-9.

### Combining dietary information and phylogenetic analysis of transcriptional regulators reveals that Unk-9 dehydroxylates HCA to support ATP production.

**Figure 3.**
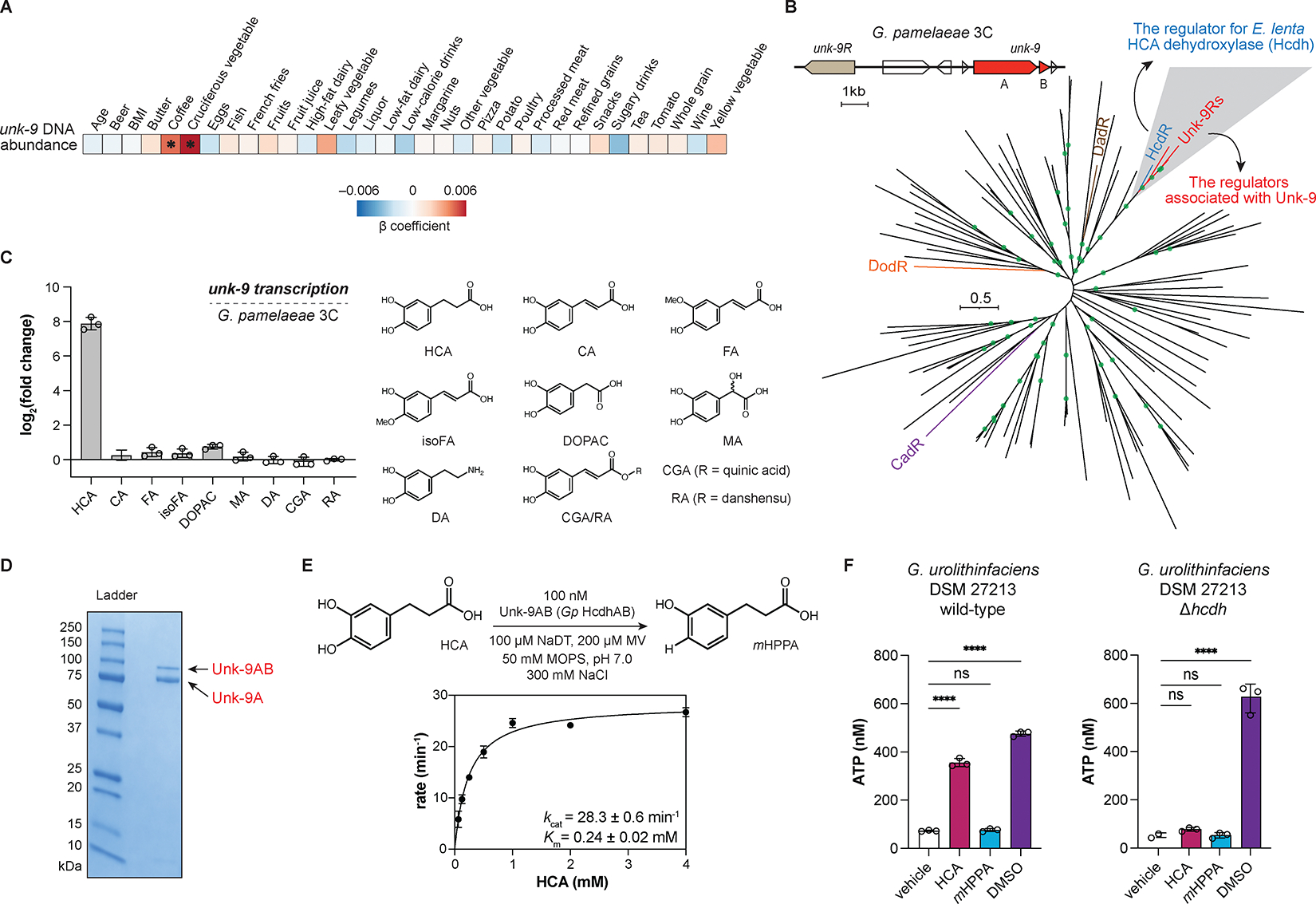
Combining dietary information and phylogenetic analysis of transcriptional regulators reveals that Unk-9 dehydroxylates HCA to support ATP production. (A) Correlation of *unk-9* DNA abundance with dietary inputs from the MLVS cohort. Linear mixed-effects model. *, FDR < 0.10. (B) Maximum-likelihood phylogenetic tree of the membrane-bound LuxR-type transcriptional regulators co-localized with molybdopterin oxidoreductases in the *E. lenta* DSM 2243 and *G. pamelaeae* 3C genomes. Branches with bootstrap number > 0.9 are highlighted by green circles. (C) Substrate-specific induction of Unk-9 expression in *G. pamelaeae* 3C by HCA analogs relative to vehicle measured by RT-qPCR. (D) SDS-PAGE gel image for isolated *Gp* Hcdh using activity-guided native purification. (E) Michaelis–Menten kinetics of *Gp* Hcdh using initial rates measured in the first 60 s. (F) ATP production in response to HCA, *m*HPPA, and DMSO in the cell suspension of *G. urolithinfaciens* DSM 27213 wild-type and Δ*hcdh* grown anaerobically with HCA. One-way ANOVA followed by Dunnett’s multiple comparisons test with all comparisons made against vehicle control. ns, not significant; ****, adjusted p-value < 0.0001. For all panels, n=3 biologically independent replicates, data presented are mean ± standard deviation. See also Figure S3, Figure S4, Table S1, and Table S4. *m*HPPA, 3-hydroxyphenyl propionic acid; MV, methyl viologen; NaDT, sodium dithionite.

The numerous potential polyphenol substrates for Unk-9 prompted us to deduce candidates using several approaches. First, we correlated Unk-9 levels in gut microbiomes with consumption of specific food groups in the MLVS cohort. We found that Unk-9 DNA abundance correlated with the long-term consumption of cruciferous vegetables and coffee, which contain high levels of HCA precursors such as chlorogenic acid (1.3–3.6 mg/g fresh weight of coffee and up to 0.7 mg/g dry weight of cruciferous vegetables) (Figure 3A and Figure S3A)^34,35^. When extended to the characterized catechol dehydroxylase clusters, this dietary analysis revealed significant positive correlations with other polyphenol-rich food groups, including fruits (Cadh) and whole grain (Dodh) (Figure S3B). No significant correlation was found between dietary inputs and Unk-9 mRNA abundance, potentially due to factors like differences between long-term diet and rapid transcriptional responses, smaller MTX dataset size, substrate availability *in vivo*, and post-transcriptional effects. Overall, this analysis suggested that polyphenols from coffee and cruciferous vegetables, such as HCA and its precursors, might be potential substrates for Unk-9.

In parallel, we used knowledge of characterized catechol dehydroxylases to generate hypotheses for the function of Unk-9. First, phylogenetic analysis of the catechol dehydroxylase catalytic subunits revealed that Unk-9 was distant from known enzymes (Figure S3C). We next examined genes that co-localize with *unk-9* in bacterial genomes because functionally associated genes are often clustered^36^. We found that *unk-9* is co-localized with a gene encoding a membrane-bound LuxR-type regulator, *unk-9R* (Figure 3B). This class of transcriptional regulators has recently been shown to regulate expression of their associated genes, including catechol dehydroxylases, in response to their specific substrates^23^. We therefore reasoned that examining the relationship of these catechol dehydroxylase regulators might provide information about their substrates. Phylogenetic analysis of this transcriptional regulator family revealed that Unk-9R was most closely related to HcdR (44% aa identity, 60% aa similarity), the regulator for *E. lenta* HCA dehydroxylase (Hcdh) (Figure 3B). This relationship is striking because their associated catechol dehydroxylases, Unk-9 and Hcdh, have low sequence identity (17% aa identity, 29% aa similarity). Altogether, by considering dietary correlations and the similarity between transcriptional regulators, we postulated Unk-9 would dehydroxylate HCA or a related analog.

To identify the precise substrate of Unk-9, we first performed RT–qPCR experiments to measure the transcriptional level of *unk-9* in *G. pamelaeae* 3C in response to HCA and related compounds. We found that HCA induced the transcription of *G. pamelaeae* 3C *unk-9* >250-fold, whereas close analogs did not induce transcription (Figure 3C). HCA dehydroxylation was observed in liquid cultures of *G. pamelaeae* 3C and several other gut Coriobacteriia strains encoding *unk-9*, some of which had previously been reported to dehydroxylate HCA (Figure S3D)^13,19^. We then obtained HCA-dehydroxylating proteins from *G. pamelaeae* 3C grown with HCA via activity-guided native purification. Proteomic analysis confirmed that the purified enzyme was Unk-9 (Figure 3D, Figure S3E, and Table S4). Assays using the purified Unk-9 enzyme, which we have named *G. pamelaeae* HCA dehydroxylase (*Gp* Hcdh), showed robust conversion of HCA to the corresponding dehydroxylated product (Figure S3F), with minimal activity toward related compounds (Figure S3H). Within the DMSO reductase superfamily, *Gp* Hcdh exhibited a similar enzyme turnover number (*k*_cat_) to perchlorate reductase (28.3 vs 27.1 min^−1^, respectively) (Figure 3E and Figure S3G)^37^. Additionally, the *K*_m_ of *Gp* Hcdh (0.24 mM) was comparable to that of nitrate reductase (0.20 mM)^37^. Inductively coupled plasma mass spectrometry (ICP-MS) analysis revealed that *Gp* Hcdh contains equimolar amounts of molybdenum relative to protein, indicating a single bis-MGD cofactor (Figure S3I).

With its biochemical function established, we next investigated the biological role of *Gp* Hcdh for the producing bacterium. Many DMSO reductase superfamily enzymes, including some catechol dehydroxylases in *Eggerthella* strains, facilitate anaerobic respiration by reducing alternative electron acceptors^13,38,39^. The similar kinetic parameters between *Gp* Hcdh and respiratory perchlorate and nitrate reductases further suggest its potential involvement in anaerobic respiration. Thus, we tested whether *Gp* Hcdh supports growth and ATP production under anaerobic conditions. We observed that HCA, but not its dehydroxylated product, promoted growth of *G. pamelaeae* 3C similarly to DMSO, another alternative electron acceptor for anaerobic respiration (Figure S4A). This growth coincided with HCA dehydroxylation (Figure S4B). Further, a *G. pamelaeae* 3C cell suspension at resting state produced ATP when incubated with HCA or DMSO (Figure S4C). To link ATP production to *Gp* Hcdh activity, we generated a Hcdh deletion mutant (Δ*hcdh*) of *G. urolithinfaciens* DSM 27213 using a recently developed CRISPR-based genetic tool for Coriobacteriia (Figure S4D)^23^. The Δ*hcdh* strain lost HCA dehydroxylase activity in culture (Figure S4E), and HCA-induced ATP production was not observed in this strain (Figure 3F). Together, these experiments support the involvement of *Gp* Hcdh in anaerobic respiration.

### Levels of *Gp* Hcdh correlate with the anti-inflammatory response to cruciferous vegetable consumption

**Figure 4.**
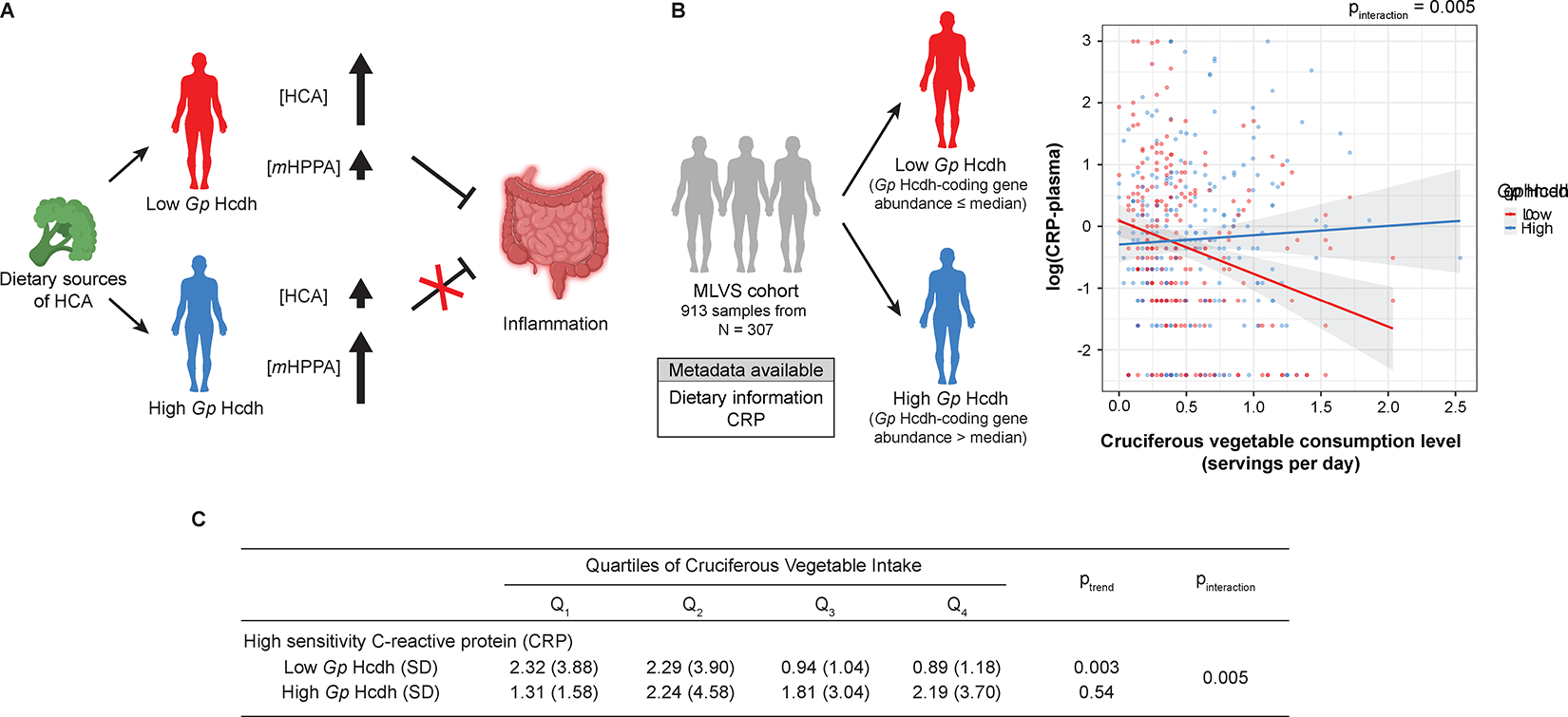
Levels of *Gp* Hcdh correlate with the anti-inflammatory response to cruciferous vegetable consumption. (A) Proposed mechanistic link between *Gp* Hcdh activity and the anti-inflammatory benefit of cruciferous vegetable consumption. (B) (left) Grouping of the MLVS cohort by *Gp* Hcdh DNA level. (right) Multivariate linear mixed model analysis for the correlation between cruciferous vegetable consumption and the level of CRP in the low (red trend line) and high (blue trend line) *Gp* Hcdh groups. Grey areas represent standard error. Panels were created using BioRender.

We next investigated potential connections between HCA dehydroxylation and host phenotypes by analyzing gut microbiome datasets. To begin, we considered the well-established anti-inflammatory activity of HCA^40^. Oral administration of HCA reduced the production of pro-inflammatory cytokines in lipopolysaccharide (LPS)-injected mice^41^, mice with liver injury^42^, and rats with dextran sulfate sodium-induced colitis^43^. HCA significantly reduced inflammatory activities in IL-1β-stimulated human colon fibroblasts^43^ and LPS-stimulated mouse and human peripheral blood mononuclear cells^44,45^. In contrast, the anti-inflammatory activity of the HCA dehydroxylation product, 3-hydroxyphenyl propionic acid (*m*HPPA), was abolished^43,44^. Therefore, we hypothesized that the dehydroxylating activity of *Gp* Hcdh might alter the anti-inflammatory benefit of consuming foods that are sources of HCA (Figure 4A).

To investigate this hypothesis, we again analyzed the MLVS data, correlating HCA dehydroxylase abundance with host inflammation markers in the context of consumption of cruciferous vegetables and coffee, which represent the main sources of HCA quantifiable by the available dietary instruments^34,35^. Given the higher mRNA abundance (33.4-fold) and prevalence (82.8% vs 19.8%) of *Gp* Hcdh compared to *E. lenta* Hcdh, we focused on *Gp* Hcdh levels and their relationship with biomarkers of host inflammation and health. The MLVS samples were divided into two groups based on their *Gp* Hcdh DNA abundance relative to the median. Within the low *Gp* Hcdh group, higher cruciferous vegetable intake was associated with reduced levels of plasma C-reactive protein (CRP), a marker of inflammation (Figure 4B and Figure 4C). Conversely, such a correlation was absent among the high *Gp* Hcdh group (p_interaction_ = 0.005). We propose this correlation could potentially be explained by the differential activity of *Gp* Hcdh within the gut microbiotas of subjects, similar to the previously reported microbiota-driven links between dietary fiber and CRP^46^. Individuals with high levels of *Gp* Hcdh may dehydroxylate HCA to a higher degree, reducing its anti-inflammatory benefits, whereas those with low levels of the enzyme might not metabolize HCA as extensively. Although mechanistic studies are needed to test causality, our results suggest a potential route by which gut microbial metabolism of a specific polyphenol might influence the impact of diet on human health.

## Discussion

It is well-established that highly expressed enzymes in microbial communities perform ecologically and metabolically important functions^29,47–50^. Though MTX can identify highly transcribed enzymes in microbial communities^24,25,28–30,51–55^, MTX-based strategies have been underutilized for enzyme discovery because of challenges in acquisition of high-quality MTX and limited examples of successful downstream biochemical characterization of the prioritized genes^24,25,53^. By integrating MTX and biochemical characterization workflows, we successfully identified a gut bacterial catechol dehydroxylase that metabolizes a common and abundant substrate. This enzyme discovery framework, coupled with the success of related previous efforts^24,25,56^, should be readily applied to other important gut bacterial enzyme families such as carbohydrate-active enzymes, sulfatases, β-glucuronidases, and glycyl radical enzymes.

A particularly challenging component of enzyme discovery workflows is linking uncharacterized sequences to their biochemical functions^24^. Catechol dehydroxylases are especially difficult in this regard because of their poorly understood substrate specificity and technical challenges associated with expressing complex metalloenzymes. We overcame these obstacles by investigating the transcriptional regulators controlling expression of catechol dehydroxylases. Individual catechol dehydroxylase genes are colocalized with genes encoding substrate-specific transcriptional regulators, enabling their association^23^. We found that the regulator of *Gp* Hcdh was most closely related to the regulator of *E. lenta* Hcdh, highlighting HCA as a potential substrate. Unlike *E. lenta* Hcdh, which has three subunits including a catalytic subunit, a subunit with four [4Fe-4S] clusters, and a membrane anchor^13^, *Gp* Hcdh has only two subunits. Their catalytic subunits are phylogenetically distant (Figure S3C), differing in length (770 aa for *Gp* Hcdh and 1193 aa for *E. lenta* Hcdh) and the presence of a twin-arginine translocation signal peptide. These differences made it challenging to deduce the biochemical function of *Gp* Hcdh from the sequence of *E. lenta* Hcdh. Our results indicate that analyses of associated regulators could be effective, underutilized strategy for assigning functions to microbial enzymes.

Finally, we uncovered a striking correlation between reduced levels of *Gp* Hcdh encoding genes in gut microbiomes and reduced levels of inflammation in subjects with high cruciferous vegetable intake. Our results may be related to previously found microbiota-driven interactions between dietary fibers or the Mediterranean diet and host health^46,57^. By uncovering a link between host inflammation and a specific gut microbial polyphenol-metabolizing enzyme, our findings set the stage for future mechanistic work to study the role of gut bacterial catechol dehydroxylation in host biology. Discovering the activities of additional polyphenol-metabolizing gut microbial enzymes and their impacts on the host and gut microbiome will be critical in deciphering the health benefits of these important dietary compounds.

### Limitations of the study

The causality of the link between *Gp* Hcdh and host CRP levels requires mechanistic validation in model organisms. Additionally, no significant interactions were observed between *Gp* Hcdh and other biomarkers of host inflammation. The MLVS cohort sampled healthy participants, so the levels of biomarkers are within a healthy range with low variation, potentially constraining the statistical analysis. Our interaction analysis only considered *Gp* Hcdh due to its high *in vivo* transcriptional levels and prevalence compared to *E. lenta* Hcdh. However, *E. lenta* Hcdh or dehydroxylases from other organisms may contribute to HCA metabolism *in vivo*.

## STAR Methods

### Resource availability

#### Lead Contact

Further information and requests for resources and reagents should be directed to and will be fulfilled by the Lead Contact, Emily P. Balskus (balskus@chemistry.harvard.edu)

#### Materials Availability

The whole genome sequence of *G. urolithinfaciens* DSM 27213 Δ*hcdh* strain generated in this study was deposited in the National Center for Biotechnology Information under the accession number of PRJNA1150218. The strain will be available directly from the authors upon reasonable request.

#### Data and Code Availability

- This paper analyzes existing, publicly available data. These accession numbers for the datasets are listed in the key resources table.
- All original codes have been deposited at Zenodo and is publicly available. DOI is listed in the key resources table.
- Any additional information required to reanalyze the data reported in this paper is available from the lead contact upon request.

### Experimental model and study participant details

#### Microbial strains

*Gordonibacter pamelaeae* 3C, *Gordonibacter sp.* 28C, *Paraeggerthella hongkongensis* RC2/2 A, and *Eggerthella lenta* strains were obtained from Peter Turnbaugh at the University of California, San Francisco. *Adlercreutzia equolifaciens* DSM 19450, *Adlercreutzia equolifaciens* subsp. *celatus* DSM 18785, *Slackia heliotrinireducens* DSM 20476, *Senegalimassilia anaerobia* DSM 25959, *Gordonibacter urolithinfaciens* DSM 27213, *Gordonibacter pamelaeae* 7–10-1b, and *Raoultibacter massiliensis* DSM 103407 were purchased from Leibniz Institute DSMZ. Unless otherwise noted, all bacterial culturing was performed in a rigid anaerobic chamber (Coy Laboratory Products) under an atmosphere of 2–4% hydrogen, 2–4% carbon dioxide, and nitrogen as the balance. Hungate tubes were used for anaerobic culture unless otherwise noted (Chemglass, catalog# CLS-4209–01). The human gut Coriobacteriia species were grown at 37 °C on BHI supplemented with 1% L-arginine monohydrochloride (w/v%), 0.05% L-cysteine hydrochloride monohydrate (w/v%), 10 mM sodium formate (BHIrcf medium) unless otherwise noted.

### Method details

#### Genome searches for catechol dehydroxylase homologs

The sequences of the catalytic subunits of the five characterized catechol dehydroxylases (Hcdh, WP_035577003.1; Dadh, WP_086414988.1; Cadh, WP_114556713.1; Dodh, WP_041239388.1; and Cldh, WP_114568826.1) were used as queries for tBLASTn search (search translated nucleotide databases using a protein query) with an e-value cutoff of <0.0001 using a MAG dataset that contains 154,723 MAGs from the human-associated microbiome^18,58^. Similar to a previous study^13^, hits with >30% aa identity to any of the queries were considered catechol dehydroxylase homologs. The cutoff was chosen because sequences with lower similarity often more closely resemble other biochemically characterized members of the DMSO reductase superfamily, as assessed by sequence identity. Since the MAG dataset contains truncated protein sequences, protein sequences shorter than 600 amino acids were filtered.

#### Phylogeny of catechol dehydroxylase-coding species

The MAGs searched in this study were previously assigned to the closest species-level genome bins (SGBs) using PhyloPhlAn 3.0^59^. For each SGB, one representative MAG was selected based on completeness and contamination. Only SGBs whose representative MAGs encode at least one catechol dehydroxylase homolog were considered catechol dehydroxylase-encoding SGBs. The representative MAGs of the catechol dehydroxylase-encoding SGBs were subjected to the construction of a maximum-likelihood phylogenetic tree using PhyloPhlAn 3.0 with the following parameters: medium diversity, fast, default PhyloPhlAn marker database, FastTree and RAxML. The representative MAG of SGB 991 was used as an outgroup to root the tree. The tree was visualized using a free web-based tool, iTOL v6.5.8^60^. SGBs were classified at family level using PhyloPhlAn 3.0 (database: SGB.Jan19). To match SGBs with cultured bacteria, the genomes of cultured Coriobacteriia were assigned to the closest SGBs using PhyloPhlAn 3.0.

#### SSN analysis of catechol dehydroxylases

The catechol dehydroxylase homologs from the MAG search were submitted to the Enzyme Function Initiative’s Enzyme Similarity Tool (EFI-EST)^22^. The alignment scores between the characterized catechol dehydroxylases were determined to range from <100 to 291 (Dadh–Cadh pair). As such, initial SSN was generated with minimum alignment scores of 300. The characterized catechol dehydroxylases were initially separated into distinct clusters with a minimum alignment score of 300 and a minimum aa sequence identity of 60%. Manual inspection revealed that the cluster containing *E. lenta* DSM 2243 Hcdh contained another sequence from *E. lenta* DSM 2243: ELEN_0497 (60.6% aa identity to Hcdh) (Figure S1B). RT–qPCR experiments showed that HCA upregulated the expression of Hcdh but not that of ELEN_0497 (Figure S1C), suggesting that ELEN_0497 likely does not metabolize HCA. To separate these two sequences, additional SSNs were generated with higher minimum aa sequence identities. ELEN_0497 was separated from the Hcdh cluster at minimum alignment score 300 and minimum aa sequence identity 72% (Figure S1B and Table S1). The final network was then downloaded as 95% representative nodes. Nodes corresponding to the five characterized dehydroxylases are enlarged. The hydroxyl group that is dehydroxylated is colored on the molecular structures. Cytoscape v.3.9.1 was used to visualize and optimize the networks^61^.

#### Catechol dehydroxylase activity assay

Turbid 48-hour starter cultures of Coriobacteriia species in BHI medium were diluted 1:100 into 200 μL of BHI medium supplemented with 10 mM sodium formate and 1 mM of a catechol substrate in triplicates. The cultures were anaerobically incubated at 37 °C for three days. For LC–MS/MS analysis of dehydroxylation activity, 40 μL of supernatant was diluted into 160 μL of LC–MS grade methanol, and the precipitates were removed by centrifugation before injection onto LC–MS/MS, as described below. In addition, the consumption of catechol substrates was assessed using a colorimetric assay (Arnow test)^62^. For the Arnow test, 25 μL of culture supernatant was sequentially added to 25 μL of 0.5 M aqueous HCl, 25 μL of an aqueous solution containing sodium molybdate (0.1 g/mL) and sodium nitrite (0.1 g/mL), and finally 25 μL of 1 M aqueous NaOH. The presence of catechol compounds was indicated by the development of a pink color, and absorbance at 500 nm was measured using a Synergy HTX Multi-Mode Microplate Reader (BioTek).

#### MGX and MTX quantification

The MGX and MTX datasets of two human studies (MLVS^28^ and HMP2^33^) were downloaded from the National Center for Biotechnology Information (MLVS: PRJNA354235, HMP2: PRJNA398089). Host-associated reads, low-quality reads, and adapter sequences were removed using KneadData v0.10.0 (default setting). To quantify the MGX and MTX abundance of individual catechol dehydroxylase clusters from the isofunctional SSN, we first used ShortBRED^63^, a highly specific tool that quantifies peptide markers unique to each cluster. However, most catechol dehydroxylase clusters were not detected using ShortBRED, presumably due to the low abundance of Coriobacteriia. Instead, to detect genes and transcripts encoding these enzymes, we quantified reads mapping on the representative sequence of each cluster. The representative sequence was chosen as the sequence that has the highest sum of alignment scores within each cluster. Singletons were excluded from the analysis because they are poorly aligned with the other sequences. To prevent non-specific read mapping, we also included UniRef50 sequences of the DMSO reductase superfamily (IPR000657) into the list (catechol dehydroxylase homologs were removed from the list manually). Using a BLASTX DIAMOND search (e-value cutoff: 0.0001)^64^, reads were mapped on the protein sequences. Reads with >72% aa identity to each sequence were counted. If a read was mapped on multiple proteins, the read was counted as a hit for the protein that had the highest identity, which increases the specificity of the quantification. The number of hits was normalized first by the number of the total reads of each sample and the lengths of the proteins to read per kilobase million (RPKM) (Table S2). For further statistical analyses across samples, RPKM was normalized by average genome size (AGS), which was calculated using MicrobeCensus from each MGX samples^65^. The plot and heatmap were generated using GraphPad PRISM 9.

#### Transcriptional analysis of *G. pamelaeae* 3C

For *in vivo* transcriptional analysis of *G. pamelaeae* 3C, Bowtie2 v2.4.2^66^ was used to map reads from quality controlled MLVS MGX and MTX data on the whole genome of *G. pamelaeae* 3C. HTseq-count v0.13.5^67^ was used to count the number of mapped reads. To account for gene copy-number variation, RNA/DNA ratio for each gene and sample was derived using paired MGX and MTX samples (Table S2). For *in vitro* transcriptional analysis, raw RNA-seq data of *G. pamelaeae* 3C in mid-exponential growth phase were downloaded from the National Center for Biotechnology Information (PRJNA450120)^14^. The downloaded RNA-seq data were processed to remove ribosomal RNA, low quality bases, and adapter sequences using KneadData v0.10.0 (--bypass-trf). The processed data were then quantified against the protein coding sequences of the strain using Salmon-quant v1.9.0 (--validateMappings)^68^. The resulting transcriptional levels of each gene were expressed in transcripts per million (TPM), which was log-transformed using the formula log_10_(TPM+1) (Table S2). The plots were generated using GraphPad PRISM 9.

#### Phylogeny of catechol dehydroxylases

The catalytic subunits of representative sequences from the catechol dehydroxylase SSN clusters were first aligned using MAFFT-linsi v7.505^69^ and then trimmed to 669 aa using trimAl v1.4.1 (-gappyout)^70^ (Table S1). A maximum-likelihood phylogenetic tree was generated using iqtree2 v2.1.3 (1000 ultrafast bootstraps)^71^. To enhance the clarity, highly divergent branches were pruned. The tree was visualized using a free web-based tool, iTOL v6.5.8^60^.

#### Phylogeny of LuxR-type transcriptional regulators

Sequences of the 10–12 transmembrane LuxR-type regulators encoded by *G. pamelaeae* 3C and *E. lenta* DSM 2243 as well as the regulators co-localized with *Gp* Hcdh homologs in the genomes of *G. pamelaeae* 7–10-1b, *G. sp.* 28C, *G. urolithinfaciens* DSM 27213, *P. hongkongensis* RC2/2 A, and *E. isourolithinifaciens* DSM 104140 were first aligned using MAFFT-linsi v7.505^69^ and then trimmed using trimAl v1.4.1 (-gappyout)^70^ (Table S1). A maximum-likelihood phylogenetic tree was generated using iqtree2 v2.1.3 (1000 ultrafast bootstraps)^71^. The tree was visualized using a free web-based tool, iTOL v6.5.8^60^.

#### RT–qPCR analysis

##### Method A (G. pamelaeae 3C)

Turbid 54-hour starter cultures of *G. pamelaeae* 3C in BHI medium were diluted 1:100 into 5 mL of BHIrcf medium in six replicates, and cultures were grown at 37 °C anaerobically. When the optical density at 600 nm (OD_600_) of the cultures reached 0.2, 50 μL of 100 mM substrate was added at a final concentration of 1 mM to three replicates, and 50 μL of vehicle (dimethylformate) was added to the other three replicates. The cultures were grown at 37 °C until they reached OD_600_ = 0.5, at which cells were pelleted by centrifugation.

##### Method B (E. lenta DSM 2243)

Turbid 54-hour grown starter cultures of *E. lenta* DSM 2243 in BHI medium were diluted 1:100 into 5 mL of BHI medium supplemented with 0.5% L-arginine-HCl (w/v%) and 10 mM sodium formate in six replicates, and cultures were grown at 37 °C anaerobically. When the cultures reached OD_600_ = 0.2, 50 μL of 100 mM substrate was added at a final concentration of 1 mM to three replicates, and 50 μL of vehicle (dimethylformate) was added to the other three replicates. The cultures were grown at 37 °C until they reached OD_600_ = 0.5, at which cells were pelleted by centrifugation.

To the bacterial pellets from all methods, 1 mL of TRIzol was added. Total RNA was isolated by bead beating for 5 min (Biospec, mini-beadbeater-12 / Zymo Research BashingBead Lysis Tubes, catalog# S6012–50) and then using the Zymo Research Direct-Zol RNA MiniPrep Plus Kit (catalog# R2070) according to the manufacturer’s protocol. Total RNA was then treated with the Promega RQ1 RNase-free DNase kit (catalog# M6101) according to the manufacturer’s protocol. RT–qPCR assays were performed using the Luna^®^ Universal One-Step RT-qPCR Kit (catalog# E3005S) with 10 ng of purified RNA using a Thermocycler CFX96 Real-Time System (Bio-Rad). The C_q_ values of target genes were normalized to those of housekeeping genes. Fold changes in transcript levels were calculated by comparing normalized C_q_ values of substrate-treated samples to those of vehicle-treated samples. The primers used for RT-qPCR experiments are listed in Table S3.

#### LC–MS/MS methods

Samples were analyzed using a Waters UPLC-QQQ Mass Spectrometer equipped with a CORTECS T3 column (Waters Corp.). The following chromatography conditions were used in all LC–MS/MS assays: Column temperature, 40 °C; Mobile phase A, water (0.1% formic acid); Mobile phase B, acetonitrile (0.1% formic acid); Gradient (percentage denotes the ratio of mobile phase A): 100%, 1 min; 100% to 10%, 1 min; 10%, 0.5 min; 10% to 100%, 0.25 min; 100%, 0.65 min; Flowrate, 0.5 mL/min; Injection volume: 1.0 μL.

For MS/MS, the precursor and daughter ions were detected via Electrospray Ionization (ESI) in negative mode. The transitions and collision energy used to detect specific compounds of interest are listed. (1) HCA: precursor ion *m/z*, 181.09; daughter ion *m/z*, 59.03; collision energy, 12 V; cone voltage, 2 V. (2) *m*HPPA: precursor ion *m/z*, 165.09; daughter ion *m/z*, 121.10; collision energy, 12 V; cone voltage, 36 V. (3) Dopamine: precursor ion *m/z*, 154.17; daughter ion *m/z*, 106.21; collision energy, 14 V; cone voltage, 58 V. (4) *m*-Tyramine: precursor ion *m/z*, 138.18; daughter ion *m/z*, 77.06; collision energy, 26 V; cone voltage, 26 V. (5) DOPAC: precursor ion *m/z*, 167.07; daughter ion *m/z*, 123.10; collision energy, 12 V; cone voltage, 46 V. (6) 3-Hydroxyphenyl acetic acid: precursor ion *m/z*, 151.08; daughter ion *m/z*, 107.10; collision energy, 8 V; cone voltage, 30 V.

#### Activity-guided purification of *Gp* Hcdh

Growth of cultures for protein purification was performed under strictly anaerobic conditions. The anaerobic atmosphere consisted of 2–4% CO_2_, 2–4% H_2_, and N_2_ as the balance. *G. pamelaeae* 3C starter cultures were inoculated from frozen glycerol stock into liquid BHI medium and were grown anaerobically without shaking for 48 hours at 37 °C. Expression medium (liquid BHI containing 25 mM L-arginine-HCl and 10 mM sodium formate) was deoxygenated under the anaerobic atmosphere for at least 24 hours, with occasional mixing. The starter cultures were then diluted 1:100 into a total volume of 8 L (4 flasks, 2 L each) of the expression medium. The expression medium was further supplemented with cysteine (6.3 mL of aqueous 1 M L-cysteine-HCl per 2 L of medium, 3 mM final concentration) and HCA (10 mL of aqueous 200 mM HCA per 2 L of medium, 1 mM final concentration). The resulting culture flasks were sealed with anaerobic septa and caps, then incubated at 37 °C for 18 hours, with shaking at 160 rpm. After 18 hours, OD_600_ usually reached ~0.6. At this point, the growth medium was analyzed for the presence of catechols. The Arnow colorimetric assay yielded a clear solution, indicating an almost complete consumption of HCA.

Activity-guided purification of *Gp* Hcdh was carried out under air, while keeping all solutions at 4 °C. Cells were pelleted by centrifugation (8k RCF, 15 min) and then suspended in lysis buffer (20 mM Tris, pH 7.5, 500 mM NaCl, 10 mM MgSO_4_, 1 mM CaCl_2_, 0.1 mg/mL DNase, 0.5 mg/mL lysozyme, 1 tablet/50 mL SIGMAFAST protease inhibitor) to give a final volume of ~45 mL. Cells were lysed via sonication (Branson Sonifier 450, 25% amplitude, 10 seconds on, 40 seconds off, 4 min total sonication time). The lysate was then clarified via centrifugation (20k RCF, 45 mins). The resulting supernatant (~40 mL) was transferred to a new 50 mL Eppendorf tube, then solid ammonium sulfate (13.2 g) was slowly added to achieve 33% w/v. The tube was inverted gently to dissolve all ammonium sulfate. It was then left to sit at 4 °C for 90 mins, with occasional inverting. The protein precipitate was pelleted by centrifugation (4k RCF, 30 mins). The precipitate was dissolved in ~25 mL of HIC-1M buffer (20 mM Tris, pH 7.5, 1 M ammonium sulfate). The protein solution was then centrifuged to remove insoluble precipitates prior to hydrophobic interaction chromatography (HIC).

The following protein purification steps were performed using an FPLC (Bio-Rad BioLogic DuoFlow System equipped with GE Life Sciences DynaLoop90). To perform HIC, the HIC column (Cytiva HiTrap Phenyl HP, 5 mL) was equilibrated with 15 mL of HIC-1M buffer. The protein solution was loaded at 2 mL/min, followed by an additional 5 mL of HIC-1M buffer. Protein purification was achieved using the following gradient of HIC-1M buffer and HIC-end buffer (20 mM Tris, pH 7.5): 100%, 5 mL; 100% to 0%, 40 mL, 0%, 15 mL (percentage denotes the ratio of HIC-1M); flowrate, 2.5 mL/min; collection volume, 1.25 mL fractions. 25 μL of each fraction was taken into an anaerobic chamber to perform an enzyme activity assay (refer to activity assays during protein purification). Fractions with activity (>10% conversion of HCA to *m*HPPA) were combined. These fractions typically eluted from the column when the mobile phase was 100% HIC-end buffer. The combined fractions were then subjected to anion exchange chromatography (AEC) without a buffer swap. Analysis of the purified fractions using sodium dodecyl-sulfate polyacrylamide gel electrophoresis showed that the HIC step was most effective in purifying the active protein.

To perform AEC, the AEC column (Cytiva Q HP, 5 mL) was equilibrated with 15 mL of HIC-end buffer. The protein solution was loaded at 2 mL/min, followed by an additional 5 mL of HIC-end buffer. Protein purification was achieved using the following gradient of HIC-end buffer and AEC-end buffer (20 mM Tris, pH 7.5, 1 M NaCl): 100%, 5 mL; 100% to 0%, 60 mL, 0%, 10 mL (percentage denotes the ratio of HIC-end); flowrate, 2.5 mL/min; collection volume, 1.25 mL fractions. 25 μL of each fraction was taken into an anaerobic chamber to perform activity assay. Fractions with high activity (complete conversion of HCA to *m*HPPA) were combined. The protein solution was concentrated, and the buffer was exchanged to give 20 mM Tris, pH 7.5, 250 mM NaCl (size-exclusion chromatography (SEC) buffer). To perform SEC, the sample was loaded onto the equilibrated SEC column (Cytiva Superdex 200 Increase 10/300 GL). Protein purification was achieved by running 2 column volume of the SEC buffer. Fractions with high activity were combined. Protein yield was 0.03 mg/L of *G. pamelaeae* 3C culture.

#### Activity assays during protein purification

Stock solutions of 2 mM HCA, 4 mM methyl viologen dichloride (MV), and 4 mM sodium dithionite (NaDT) were prepared in buffer containing 20 mM Tris, pH 7.5, 250 mM NaCl under anaerobic atmosphere prior to the activity assay. The following assays were set up and conducted in the rigid anaerobic chamber (Coy Laboratory Products). To perform the enzyme activity assay, 25 μL of enzyme solution was added to 2 mM HCA solution (25 μL, 500 μM final substrate concentration) with mixing. This was followed by the addition of 4 mM MV (25 μL, 1 mM final concentrations) and 4 mM NaDT (25 μL, 1 mM final concentrations). The solution typically turns purple after the addition of NaDT. The final solution was sealed and left at room temperature, without shaking, for 20–24 hours. The assay mixture was analyzed using the Arnow colorimetric assay and LC–MS/MS. For the colorimetric assay, 25 μL of the solution was used directly. For LC–MS/MS, 20 μL of the solution was first diluted 1:10 into LC–MS grade methanol, followed by centrifugation to remove precipitates. The supernatant was diluted 1:5 into deionized water prior to analysis.

#### Proteomics analysis

SDS-PAGE was carried out with a Novex 10–20% Tris-Glycine Mini Protein Gel in Tris-Glycine-SDS buffer (100 V for 20 minutes, followed by 140 V for 50 min, room temperature). Protein samples were prepared with traditional Laemmli buffer without heating. The Precision Plus Protein All Blue Prestained Protein Standards (Bio-Rad) was used as a ladder. The gel band corresponding to the predicted molecular weight of *Gp* Hcdh was excised and submitted for proteomic analysis. The downstream experiments (sample preparation, mass spectrometry, and MS data analysis) were performed by the Harvard Center for Mass Spectrometry.

##### Sample preparation

The cut-out gel band was washed twice with 50% aqueous acetonitrile for 5 mins followed by drying in a SpeedVac. The gel was then added to a volume of 100 μL of buffer containing 20 mM tris(2-carboxyethyl)phosphine (TCEP) in 25 mM tetraethylammonium bromide (TEAB) at 37 °C for 45 minutes. After cooling to room temperature, the TCEP solution was removed and replaced with the same volume of 10 mM iodoacetamide Ultra (Sigma) in 25 mM TEAB and kept in the dark at room temperature for 45 mins. Gel pieces were washed with 200 μL of 100 mM TEAB (10 minutes). The gel pieces were then shrunk with acetonitrile. The liquid was then removed followed by swelling with the 100 mM TEAB again and dehydration/shrinking with the same volume of acetonitrile. All liquid was removed, and the gel was completely dried in a SpeedVac for ~20 minutes. 0.06 μg/5 μL of trypsin in 50 mM TEAB was added to the gel pieces and the resulting mixture was placed in a thermomixer at 37 °C for about 15 minutes. 50 μL of 50 mM TEAB was added to the gel slices. The samples were vortexed, centrifuged, and placed back in the thermomixer at 37 °C for digestion. Peptides were extracted with 50 μL of 20 mM TEAB for 20 minutes and 1 change of 50 μL of 5% formic acid in 50% acetonitrile at room temperature for 20 minutes while in a sonicator. All extracts obtained were pooled into an HPLC vial and were dried using a SpeedVac to the desired volume (~50 μL). This sample was used for protein identification by LC–MS/MS, as described below.

##### Mass spectrometry

Each sample was submitted for a single LC–MS/MS experiment that was performed on an Orbitrap Elite Hybrid Ion Trap-Orbitrap Mass Spectrometer (Thermo Fischer, San Jose, CA) equipped with Waters Aquity with nanopump and nanoLC (Waters Corp. Milford, MA). Peptides were separated using a 100 μm inner diameter microcapillary trapping column packed first with approximately 5 cm of C18 Reprosil resin (5 μm, 100 Å, Dr. Maisch GmbH, Germany) followed by a Waters analytical column. Separation was achieved through applying a gradient from 5–27% acetonitrile with 0.1% formic acid over 90 minutes at 200 nL/min. Electrospray ionization was enabled through applying a voltage of 1.8 kV using a home-made electrode junction at the end of the microcapillary column. The Elite Orbitrap Velos was operated in data-dependent mode for the mass spectrometry methods. The mass spectrometry survey scan was performed in the Orbitrap in the range of 395–1,800 *m/z* at a resolution of 6 × 10^4^, followed by the selection of the thirty most intense ions (TOP30) for CID-MS2 fragmentation in the ion trap using a precursor isolation width window of 2 *m/z*, AGC setting of 10,000, and a maximum ion accumulation of 200 ms. Singly charged ion species were not subjected to CID fragmentation.

##### MS data analysis

Raw data were submitted for analysis using Proteome Discoverer 2.4 (Thermo Scientific) software. Assignment of MS/MS spectra was performed using the Sequest HT algorithm by searching the data against a protein sequence database including all entries from the users provided database as well as the Uniprot-Gordonibacter+pamelaeae-filtered-organism_2020.fasta as well as other known contaminants such as human keratins and common lab contaminants. Sequest HT searches were performed using a 20 ppm precursor ion tolerance and requiring each peptides N/C-termini to adhere with Trypsin protease specificity, while allowing up to two missed cleavages. A MS2 spectra assignment false discovery rate (FDR) of 1% on both protein and peptide level was achieved by applying the target-decoy database search. Filtering was performed using a Percolator. Peptide N termini and lysine residues (+229.162932 Da) were set as static modifications while methionine oxidation (+15.99492 Da) was set as variable modification. A MS2 spectra assignment false discovery rate (FDR) of 1% on both protein and peptide level was achieved by applying the target-decoy database search. Filtering was performed using a Percolator (64-bit version).

#### Determination of enzyme kinetics

All assay concentrations are final concentrations. Anoxic *Gp* Hcdh and solid substrates were brought into an anaerobic chamber containing N_2_ and <0.1 ppm O_2_ (MBRAUN). Substrates were dissolved in pre-reduced dimethyl formate. Assays were setup by adding 100 nM *Gp* Hcdh to an anoxic buffer (50 mM MOPS, pH 7.0, 300 mM NaCl) containing 200 μM MV, 100 μM NaDT, and 2-fold serial dilutions of HCA (0.0625 to 4 mM). Initial 1 minute rate was measured using a continuous spectrophotometric assay. In this assay, the reductive dehydroxylation of *Gp* Hcdh was coupled with the oxidation of MV^+^ (blue, absorption maxima at 605 nm wavelength) to MV^2+^ (colorless). Each assay was performed in technical triplicates. Fitted parameters are mean ± standard error (n=3) as derived from nonlinear curve fitting to the Michaelis–Menten equation.

#### Bacterial growth assay

Turbid 48-hour starter cultures of *G. pamelaeae* 3C in BHI medium were diluted 1:100 into 1 mL of 50% BHI medium containing 10 mM sodium formate and an additive (vehicle, 5 mM HCA, 5 mM *m*HPPA, or 14 mM DMSO) in triplicates, which were arrayed into a deep-well 96-well plate (Corning, P-2ML-SQ-C-S). The plate was then sealed and anaerobically incubated at 37 °C for three days. 200 μL of aliquots were taken every 24 hours. The growth was assessed by OD_600_ measurement on a 96-well plate using a Synergy HTX Multi-Mode Microplate Reader (BioTek). To assess dehydroxylation activity, 20 μL of each aliquot was diluted into 180 μL of LC–MS grade methanol, and the precipitates were removed by centrifugation before injection onto LC–MS/MS. HCA and *m*HPPA were quantified using LC–MS/MS as described above.

#### ATP quantification assay

Turbid 54-hour starter cultures of *G. pamelaeae* 3C, *G. urolithinfaciens* wild-type, or Δ*hcdh* strain in BHI medium were diluted 1:100 into 5 mL of BHI medium supplemented with 10 mM sodium formate in triplicates, and cultures were grown at 37 °C anaerobically for 20 hours. The following steps were then performed under anaerobic conditions. Cells were pelleted by centrifugation, and the supernatant was removed. The cell pellets were washed and resuspended with 0.5 mL of pre-reduced phosphate buffer (40 mM potassium phosphate, 10 mM magnesium chloride, 10 mM sodium formate, pH 7.0). The cell suspensions were incubated in the presence of 1 mM HCA, 1 mM *m*HPPA, 14 mM DMSO, or vehicle at room temperature for 1 hour. The level of ATP in cell suspensions was quantified using PROMEGA BacTiter-Glo (catalog# 8230) and a Synergy HTX Multi-Mode Microplate Reader (BioTek).

#### *Gp* Hcdh knock out using a CRISPR gene editing tool

Plasmid construction was carried out using standard molecular biology techniques, and primers used for plasmid construction are listed in Table S3. To construct an entry plasmid for gene deletion in *G. urolithinfaciens* DSM 27213, we first introduced tandem terminators flanking expression cassette to a previously reported vector pXD68Kan2 (https://www.addgene.org/191248/). Plasmid pXD68Kan2 backbone was first amplified using primers oXD855 and oXD856 and was ligated to a fragment containing tandem terminators amplified from pXD71Cas10RFP (https://www.addgene.org/192273/) using primers oXD859 and oXD860. The resulting plasmid was then amplified with primers oXD291 and oXD292 to provide plasmid backbone containing terminators flanking expression cassette.

Primers oXD1081 and oXD1181 were used to amplify RFP insert from pXD71Cas10RFP and introduce the flanking *G. urolithinfaciens* CRISPR repeats. Primers oXD361 and oXD1179 were used to amplify a cumate-inducible genetic part from plasmid pXD70CT3. These two fragments were cloned to pXD68Kan2 backbone to make an entry CRISPR plasmid pXD80GuCas3.0RFP. To make *Gp* Hcdh-targeting plasmid pXD80GuCas3.1, oXD889 and oXD890 were annealed and phosphorylated and were introduced to pXD80GuCas3.0RFP in replace of the RFP insert using Golden Gate assembly following a previously reported method^23^.

*G. urolithinfaciens* DSM 27213 electrocompetent cells were prepared according to a previously reported method^23^. Briefly, a turbid 48-hour starter culture of each strain in BHIrcf medium was inoculated 1:50 into 40 mL of BHI medium supplemented with 1% L-arginine monohydrochloride (w/v%). When the culture reached OD_600_ of 0.3, the cells were pelleted by centrifugation, and the supernatant was removed. The cell pellet was washed with 10 mL of ice-cold sterile water three times and then with 5 mL of deoxygenated sterile 10% (v/v) glycerol aqueous solution. Finally, the cell pellet was resuspended in 2.5 mL of deoxygenated sterile 10% (v/v) glycerol aqueous solution and subdivided into 100 μL aliquots, which were flash frozen and stored at −80 °C until use.

For transformation, electrocompetent *G. urolithinfaciens* cells were electroporated using a MicroPulser Electroporator (BioRad) with 1,000 ng of pXD80GuCas3.1 at 2.5 kV voltage in 1-mm gap width electroporation cuvettes (VWR). 1 mL of pre-reduced BHIrcf medium was immediately added to the electroporated cells and transferred to 1.7 mL Eppendorf tubes. These tubes were brought to anaerobic chamber (Coy Laboratory Products) and incubated at 37 °C for 3 h. The cultures were then plated onto BHI agar plates supplemented with 100 μg/mL kanamycin, 1% L-arginine monohydrochloride (w/v%), and 10 mM sodium formate and grown anaerobically for 3–5 days at 37 °C. Single colonies were picked and inoculated into BHIrcf medium containing 100 μg/mL kanamycin, and grown for 2 days at 37 °C. Each saturated culture was then serially 10-fold diluted onto BHI agar plates supplemented with 100 μg/mL kanamycin, 1% L-arginine-HCl (w/v%), and 10 mM sodium formate with or without 50 μM inducer cumate and grown for 3–4 days. The surviving colonies of pXD80GuCas3.1 on cumate-containing plates were then analyzed individually using colony PCR and Sanger sequencing to confirm the presence of desired mutations. A single clone that contains desired mutation on *Gp* Hcdh was inoculated into BHIrcf medium containing 100 μg/mL kanamycin to get *G. urolithinfaciens* Δ*hcdh* strain.

To cure plasmid from the *G. urolithinfaciens* Δ*hcdh* strain, the strain was passaged in the absence of antibiotics for multiple rounds. The strain was inoculated into BHIrcf medium without antibiotics and let grow for 48 h. The saturated cultures were first inoculated 1:1000 into BHIrcf medium, and then the resulting diluted cultures were further diluted 1:1000 into BHIrcf medium. The 1:10^6^ diluted cultures were grown in the absence of antibiotics anaerobically for 2 days. The saturated cultures were then serially 10-fold diluted onto BHI agar plates supplemented with 1% L-arginine monohydrochloride (w/v%), 10 mM sodium formate, and with or without 100 μg/mL kanamycin and grown for 2–3 days, and CFU counts were performed. Each culture was passaged 2–3 times using the procedure described above to quantify plasmid maintenance. Lastly, single colonies were picked from BHI agar plates supplemented with 1% L-arginine monohydrochloride (w/v%), 10 mM sodium formate without kanamycin to BHIrcf medium with or without 100 μg/mL kanamycin to confirm sensitivity to kanamycin.

### Quantification and statistical analysis

#### Diet analysis

##### Study population and stool sample collection

The Men’s Lifestyle Validation Study (MLVS) consisted of 914 men aged 45 to 80 years and free from coronary heart disease, stroke, cancer, or major neurological disease at recruitment in 2011. The MLVS study population was recruited from the Health Professionals Follow-up Study (HPFS), an ongoing prospective cohort study of 51,529 US male health professionals initiated in 1986^72^. From 2011 to 2013, 307 participants provided up to two pairs of self-collected stool samples in the MLVS as detailed elsewhere^28^. In addition, participants provided two fasting blood samples twice, six months apart, during the same period as fecal samples collection as detailed elsewhere^57^. High-sensitive C-reactive protein concentrations were determined using an immunoturbidimetric high sensitivity assay using reagents and calibrators from Denka Seiken (Niigata, Japan) with assay day-to-day variability between 1 and 2%.

##### Dietary assessment

In 2012, the MLVS administered two semi-quantitative food frequency questionnaires (SFFQs) developed by Willett and his colleagues^73^ 6-months apart to collect dietary information. Participants reported their usual dietary intake (from never to ≥6 times per day) of a standard portion size (e.g., 0.5 cup of strawberries, 1 banana and 0.5 cup of cooked spinach) over the preceding year / 6 months on each SFFQ. Frequencies and portions of each individual food item were converted to average daily intake for each participant. The reproducibility and validity of these SFFQs in measuring dietary intake have been documented in detail^73–76^. For this analysis, to minimize measurement error, we calculated cumulative average dietary intake for each participant across the two SFFQs.

##### Statistical analysis

For per-feature tests between relative abundance of each gene and specific food groups and/or dietary patterns, we leveraged linear mixed effects model in the R package MaAsLin2 1.0.0^77^. All the MaAsLin2 models included identifier of participant as random effects and exposure variable and covariables (age, body mass index [BMI, kg/m2], recent antibiotic use, and Bristol stool score) as fixed effects, with the following formula:

enzymeabundance~foodconsumption+diet+BMI+age+Bristolscore+recentantibiotics+(1|participant)

To test the interaction between food intakes and CRP, we applied linear mixed models that include a product term of the food groups and each dichotomized microbial enzyme (e.g., *Gp* Hcdh > median: *Gp* Hcdh_binary = 1, *Gp* Hcdh ≤ median: *Gp* Hcdh_binary = 0), in addition to their main effects, as below.

log(crp_plasma)~cruciferousveg.intake+GpHcdh_binary+cruciferousveg.intake*GpHcdh_binary+age+BMI+recentantibiotics+Bristolscore+coffeeintake+(1|participant)

The $p_{interaction}$ was derived from the product term. These linear mixed models also include identifier of participant as random effects and simultaneously for covariables as fixed effects. Unless otherwise noted, all the p-values were corrected for multiple comparisons using the Benjamini-Hochberg procedure with a q threshold of 0.10.

## Supplementary Material

1 Document S1. Figure S1–S4

2 Table S1: Protein sequences and multiple sequence alignments, related to Figure 1 and Figure 3

3 Table S2: MGX-MTX quantification results for SSN, related to Figure 2

4 Table S3: Oligonucleotides used in this study, related to STAR Methods

5 Table S4: Proteomics IDs from activity-guided protein purification, related to Figure 3

## Figures and Tables

**Key resources table:** 

REAGENT or RESOURCE	SOURCE	IDENTIFIER
Bacterial and virus strains		
*Gordonibacter pamelaeae* 3C	Prof. Peter Turnbaugh	N/A
*Gordonibacter sp.* 28C	Prof. Peter Turnbaugh	N/A
*Paraeggerthella hongkongensis* RC2/2A	Prof. Peter Turnbaugh	N/A
*Eggerthella lenta* A2	Prof. Peter Turnbaugh	N/A
*Eggerthella Sinensis* DSM 16107	Prof. Peter Turnbaugh	N/A
*Adlercreutzia equolifaciens* DSM 19450	DSMZ	N/A
*Adlercreutzia equolifaciens* subsp. *celatus* DSM 18785	DSMZ	N/A
*Slackia heliotrinireducens* DSM 20476	DSMZ	N/A
*Senegalimassilia anaerobia* DSM 25959	DSMZ	N/A
*Gordonibacter urolithinfaciens* DSM 27213	DSMZ	N/A
*Gordonibacter pamelaeae* 7-10-1b	DSMZ	N/A
*Raoultibacter massiliensis* DSM 103407	DSMZ	N/A
*Gordonibacter urolithinfaciens* DSM 27213 *hcdh* K/O	This paper	N/A
*Gordonibacter sp.* 28C	Prof. Peter Turnbaugh	N/A
*Paraeggerthella hongkongensis* RC2/2A	Prof. Peter Turnbaugh	N/A
*Eggerthella lenta* A2	Prof. Peter Turnbaugh	N/A
*Eggerthella Sinensis* DSM 16107	Prof. Peter Turnbaugh	N/A
*Adlercreutzia equolifaciens* DSM 19450	DSMZ	N/A
*Adlercreutzia equolifaciens* subsp. *celatus* DSM 18785	DSMZ	N/A
*Slackia heliotrinireducens* DSM 20476	DSMZ	N/A
*Senegalimassilia anaerobia* DSM 25959	DSMZ	N/A
*Gordonibacter urolithinfaciens* DSM 27213	DSMZ	N/A
*Gordonibacter pamelaeae* 7-10-1b	DSMZ	N/A
*Raoultibacter massiliensis* DSM 103407	DSMZ	N/A
*Gordonibacter urolithinfaciens* DSM 27213 *hcdh* K/O	This paper	N/A
Chemicals, peptides, and recombinant proteins		
3,4-dihydroxyhydrocinnamic acid (hydrocaffeic acid; HCA)	Sigma-Aldrich	Cat#102601-10G
3-(3-hydroxyphenyl)propionic acid (*m*HPPA)	Toronto Research Chemicals	Cat#H940090-1g
Caffeic acid	Millipore Sigma	Cat#C0625-2G
3,4-dihydroxyphenylacetic acid (DOPAC)	Millipore Sigma	Cat#850217-1G
Dopamine	Sigma-Aldrich	Cat#PHR1090-1G
Chlorogenic acid	Ambeed	Cat#A163338
*trans*-ferulic acid	Sigma-Aldrich	Cat#128708-5G
*trans*-isoferulic acid	Sigma-Aldrich	Cat#PHL89717-100MG
DL-3,4-dihydroxymandelic acid	Sigma-Aldrich	Cat#151610-500MG
Rosmarinic acid	Astatech	Cat#44324
L-arginine monohydrochloride	Sigma-Aldrich	Cat#A5131-500G
L-cysteine hydrochloride monohydrate	Sigma-Aldrich	Cat#C7880-100G
Sodium formate	Sigma-Aldrich	Cat#247596-100G
Methyl viologen	Sigma-Aldrich	Cat#856177-1g
Sodium dithionite	Sigma-Aldrich	Cat#157953-5G
Ammonium sulfate	Sigma-Aldrich	Cat#157953-5G
Sodium molybdate	Sigma-Aldrich	Cat#243655-100G
Sodium nitrite	Sigma-Aldrich	Cat#237213-100G
SIGMAFAST protease inhibitor tablets	Sigma-Aldrich	Cat#S8830
DNase	Sigma-Aldrich	Cat#DN25-1G
Lysozyme	Sigma-Aldrich	Cat#L6876-5G
Brain–Heart Infusion (BHI)	Becton Dickinson	Cat#211060
TRIzol	Invitrogen	Cat#15596026
3-(3-hydroxyphenyl)propionic acid (*m*HPPA)	Toronto Research Chemicals	Cat#H940090-1g
Caffeic acid	Millipore Sigma	Cat#C0625-2G
3,4-dihydroxyphenylacetic acid (DOPAC)	Millipore Sigma	Cat#850217-1G
Dopamine	Sigma-Aldrich	Cat#PHR1090-1G
Chlorogenic acid	Ambeed	Cat#A163338
*trans*-ferulic acid	Sigma-Aldrich	Cat#128708-5G
*trans*-isoferulic acid	Sigma-Aldrich	Cat#PHL89717-100MG
DL-3,4-dihydroxymandelic acid	Sigma-Aldrich	Cat#151610-500MG
Rosmarinic acid	Astatech	Cat#44324
L-arginine monohydrochloride	Sigma-Aldrich	Cat#A5131-500G
L-cysteine hydrochloride monohydrate	Sigma-Aldrich	Cat#C7880-100G
Sodium formate	Sigma-Aldrich	Cat#247596-100G
Methyl viologen	Sigma-Aldrich	Cat#856177-1g
Sodium dithionite	Sigma-Aldrich	Cat#157953-5G
Ammonium sulfate	Sigma-Aldrich	Cat#157953-5G
Sodium molybdate	Sigma-Aldrich	Cat#243655-100G
Sodium nitrite	Sigma-Aldrich	Cat#237213-100G
SIGMAFAST protease inhibitor tablets	Sigma-Aldrich	Cat#S8830
DNase	Sigma-Aldrich	Cat#DN25-1G
Lysozyme	Sigma-Aldrich	Cat#L6876-5G
Brain–Heart Infusion (BHI)	Becton Dickinson	Cat#211060
TRIzol	Invitrogen	Cat#15596026
Critical commercial assays		
Direct-Zol RNA MiniPrep Plus Kit	Zymo Research	Cat#R2070
RQ1 RNase-free DNase kit	Promega	Cat#M6101
Luna^®^ Universal One-Step RT-qPCR Kit	New England Biolabs	Cat#E3005S
BacTiter-Glo	Promega	Cat#8230
RQ1 RNase-free DNase kit	Promega	Cat#M6101
Luna^®^ Universal One-Step RT-qPCR Kit	New England Biolabs	Cat#E3005S
BacTiter-Glo	Promega	Cat#8230
Deposited data		
*Gordonibacter urolithinfaciens* DSM 27213 *hcdh* K/O genome	This work	BioProject PRJNA1150218
Metagenome-assembled genomes from human-associated microbiome	Pasolli et al.	N/A
*Gordonibacter pamelaeae* 3C RNA-seq raw data	Bess et al.	BioProject PRJNA450120
Men’s Lifestyle Validation Study (MLVS)	Mehta et al.	BioProject PRJNA354235
Human Microbiome Project 2 (HMP2)	Lloyd-Price et al.	BioProject PRJNA398089
Recombinant DNA		
pXD71Cas10RFP	Addgene	Plasmid #192273
pXD68Kan2	Addgene	Plasmid #191248
pXD80GuCas3.1	This work	N/A
Software and algorithms		
GraphPad Prism 9 and 10	GraphPad Software	N/A
tblastn	Altschul et al.	N/A
PhyloPhlAn 3.0	Asnicar et al.	https://github.com/biobakery/phylophlan
iTOL v6.5.8	Letunic and Bork	https://github.com/iBiology/iTOL
Enzyme Function Initiative’s Enzyme Similarity Tool (EFI-EST)	Zallot et al.	https://efi.igb.illinois.edu/efi-est/
Cytoscape v3.9.1	Shannon et al.	https://github.com/cytoscape
KneadData v0.10.0	N/A	https://github.com/biobakery/kneaddata
ShortBRED	Kaminski et al.	https://github.com/biobakery/shortbred
DIAMOND	Buchfink et al.	https://github.com/bbuchfink/diamond
MicrobeCensus	Nayfach and Pollard	https://github.com/snayfach/MicrobeCensus
Bowtie2 v2.4.2	Langmead and Salzberg	https://bowtie-bio.sourceforge.net/bowtie2/index.shtml
HTseq-count v0.13.5	Anders et al.	https://htseq.readthedocs.io/en/release_0.11.1/index.html
Salmon v1.9.0	Patro et al.	https://combine-lab.github.io/salmon/
MaAsLin2 1.0.0	Mallick et al.	https://github.com/biobakery/Maaslin2
MAFFT-linsi v7.505	Katoh and Standley	https://mafft.cbrc.jp/alignment/software/
trimAl v1.4.1	Capella-Gutiérrez et al.	https://vicfero.github.io/trimal/
iqtree2 v2.1.3	Minh et al.	https://github.com/iqtree/iqtree2
Diet and MTX analysis pipeline	This paper	https://zenodo.org/records/13390717

## Highlights

- Metatranscriptomics analysis identifies prevalent gut bacterial catechol dehydroxylases
- A catechol dehydroxylase from *G. pamelaeae (Gp* Hcdh) is highly transcribed *in vivo*
- *Gp* Hcdh dehydroxylates hydrocaffeic acid, an abundant anti-inflammatory polyphenol
- *Gp* Hcdh level correlates with anti-inflammatory activity of cruciferous vegetables
