# Supplementary material for: Metatranscriptomics-guided discovery and characterization of a polyphenol-metabolizing gut microbial enzyme
Source: Cell Host Microbe. Author manuscript; Available in PMC 2024 Nov 23. (PMC11585353; doi:10.1016/j.chom.2024.10.002)
Supplement: 1 — Document S1. Figure S1–S4 [file NIHMS2033475-supplement-1.pdf]

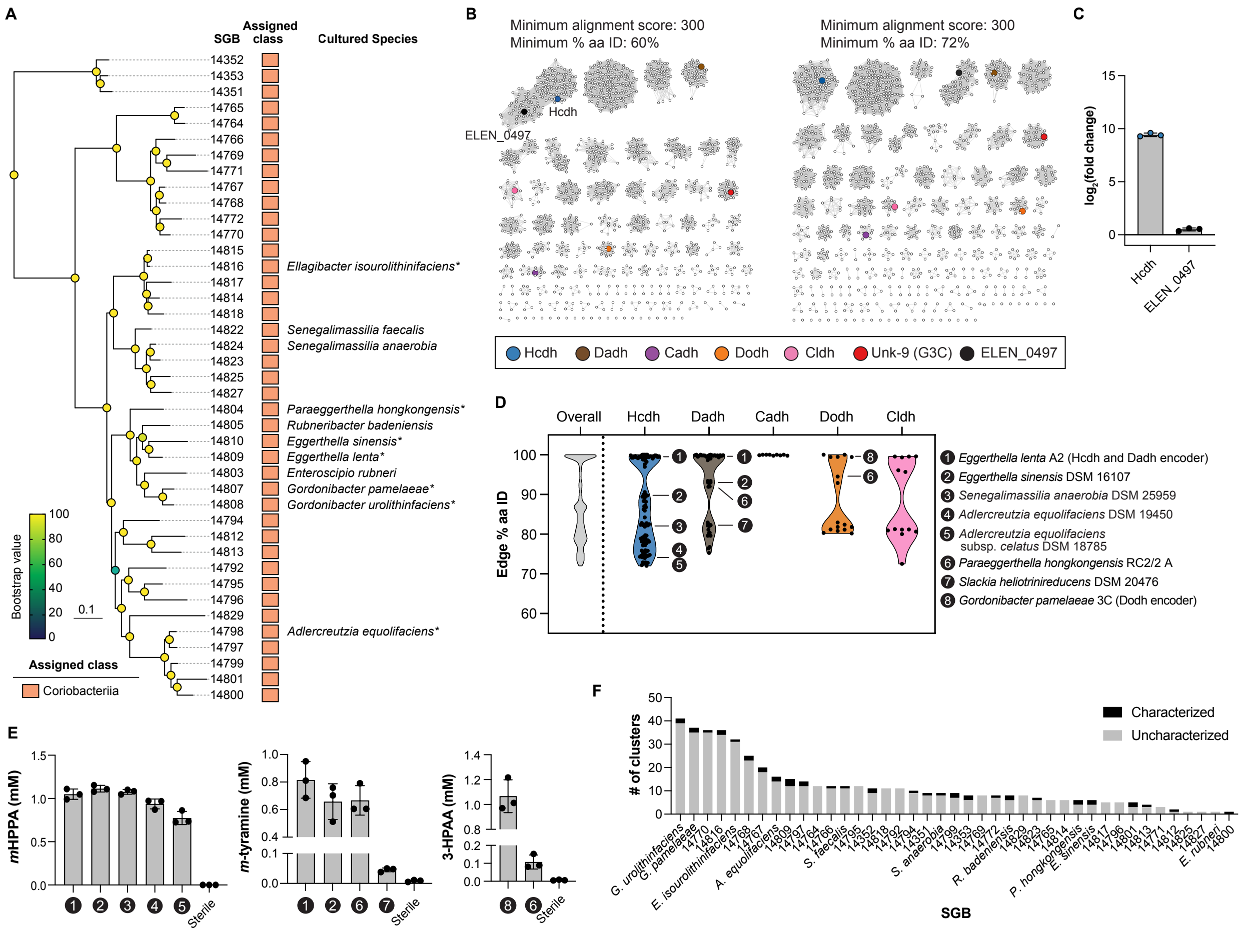

Figure S1. MAG search and generation of SSNs of human gut catechol dehydroxylases, related to Figure 1.

(A) The maximum likelihood phylogenetic tree of catechol dehydroxylase-encoding species-level genome bins (SGBs). The representative MAG of SGB 991, a human-associated Coriobacteriia species that does not encode catechol dehydroxylase homologs, was used as an outgroup to root the tree. Each cultured SGB was matched with the name of a known species. *, cultured species that were reported to have catechol dehydroxylating activity. (B) SSNs of human gut catechol dehydroxylase homologs with varying levels of the minimum alignment score and sequence identity. The nodes corresponding to the five characterized catechol dehydroxylases, ELEN_0497, and Unk-9 (*G. pamelaeae* 3C) are colored. Each node represents sequences with >95% aa ID (C) Transcriptional response of *E. lenta* DSM 2243 genes encoding Hcdh or ELEN_0497 to 1 mM HCA relative to vehicle determined by quantitative reverse-transcription PCR (RT–qPCR) (n=3 biologically independent replicates, data presented are mean ± standard deviation). (D) Identification of cultured bacteria that encode sequences in the Hcdh, Dadh, and Dodh clusters. (E) Dehydroxylating activity of the Hcdh, Dadh, and Dodh homologs-encoding species. *m*HPPA, *m*-tyramine, and 3-HPAA are the dehydroxylated products of Hcdh, Dadh, and Dodh, respectively. The dehydroxylated metabolites were quantified by LC–MS/MS. Bacterial cultures were grown with 1 mM substrates for 72 hours at 37 °C (n=3 biologically independent replicates, data presented are mean ± standard deviation). (F) The number of characterized and uncharacterized SSN clusters encoded by each SGB. SGB, species-level genome bin; DOPAC, 3,4-dihydroxyphenylacetic acid; *m*HPPA, 3-hydroxyphenyl propionic acid; 3-HPAA, 3-hydroxyphenylacetic acid.

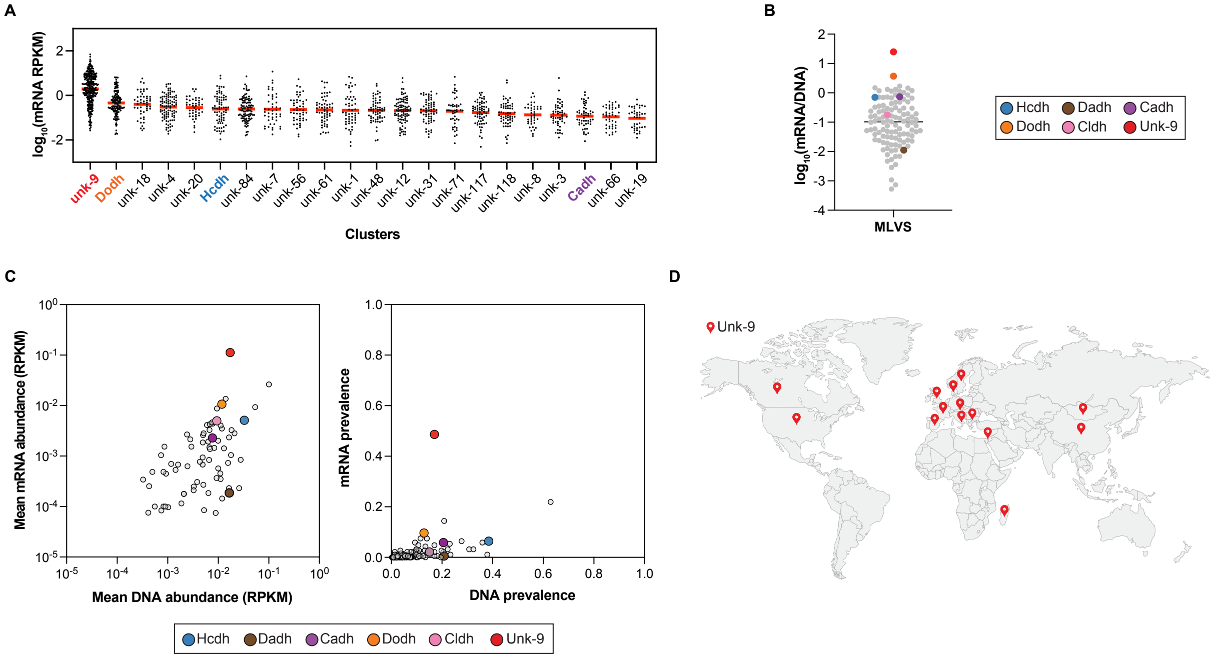

Figure S2. Quantitative MGX and MTX analysis of catechol dehydroxylase clusters, related to Figure 2. (A) The mRNA abundance of each catechol dehydroxylase clusters per individual in the MLVS dataset (clusters with prevalence > 0.1). (B) Average mRNA/DNA ratio for each catechol dehydroxylase cluster among the MLVS cohort. Characterized clusters and Unk-9 cluster are highlighted by color. Line represents the median. (C) Analysis of DNA and mRNA abundance and prevalence of catechol dehydroxylase clusters using the HMP2 datasets (healthy participants). (D) Global distribution of Unk-9 sequences in MAGs reconstructed from human fecal metagenomic samples. The countries from which Unk-9 encoding MAGs were sampled are indicated with red marks.

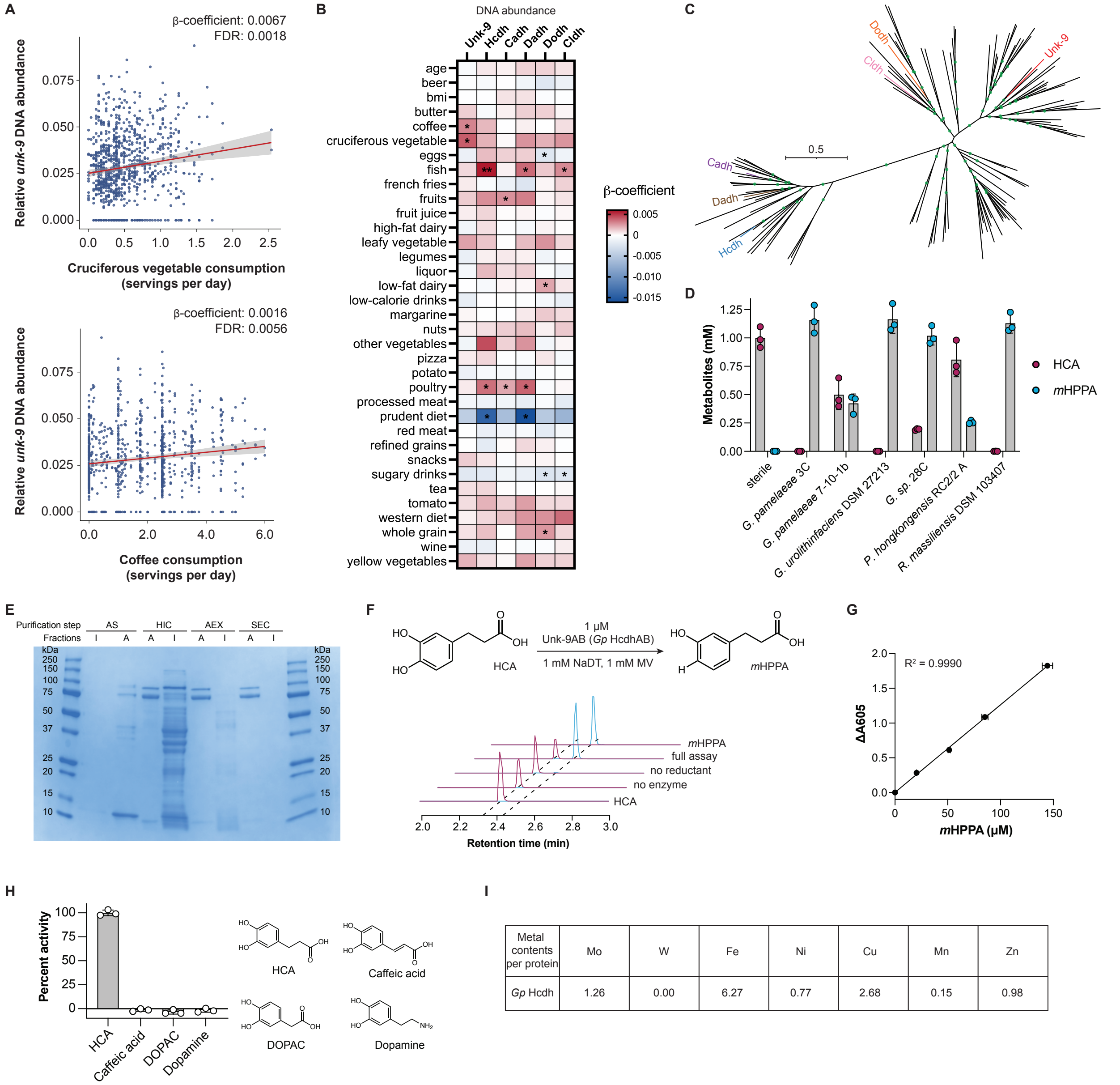

I

Metal contents per protein	Mo	W	Fe	Ni	Cu	Mn	Zn
Gp Hcdh	1.26	0.00	6.27	0.77	2.68	0.15	0.98

Figure S3. Characterization of Unk-9 enzyme function, related to Figure 3.

(A) Correlation between the DNA abundance of *Gp* Hcdh and cruciferous vegetable consumption (top panel) and coffee consumption (bottom panel). Linear mixed-effects model. (B) Correlations between diet and DNA levels of genes encoding the five characterized catechol dehydroxylases and Unk-9 in gut metagenomes. Linear mixed-effects model. *, q < 0.10; **, q < 0.01 (C) A maximum-likelihood phylogenetic tree of catechol dehydroxylases in the human gut. Branches with bootstrap number > 0.9 are marked by green circle. (D) The HCA dehydroxylation activity assay of Unk-9 encoding species (n=3 biologically independent replicates, data presented are mean ± standard deviation). (E) SDS-PAGE image of active and inactive fractions after each purification step of *Gp* Hcdh. AS, ammonium sulfate precipitation; HIC, hydrophobic interaction column; AEX, anionic exchange column; SEC, size exclusion column; A, active fraction; I, inactive fraction. (F) The *in vitro* activity assays of *Gp* Hcdh. LC–MS/MS mass chromatograms of HCA (*m/z* 181.09 → 59.03, red line) and *m*HPPA (*m/z* 165.09 → 121.10, blue line). 500 μM of HCA was incubated with 1 μM of the purified enzyme, 1 mM MV, and 1 mM NaDT in 250 mM NaCl, 20 mM Tris pH 7.0 buffer at room temperature for 24 hours. (G) Linear correlation between absorbance at 605 nm wavelength and the production of dehydroxylated *m*HPPA quantified by LC–MS/MS. (H) Substrate

specificity of *Gp* Hcdh. Percent activity was calculated by comparing 1 min initial rates to that of HCA. (2 mM substrate, room temperature, 100 nM *Gp* Hcdh, pH 7.0 50 mM MOPS buffer, 300 mM NaCl). (I) Metal contents of *Gp* Hcdh.

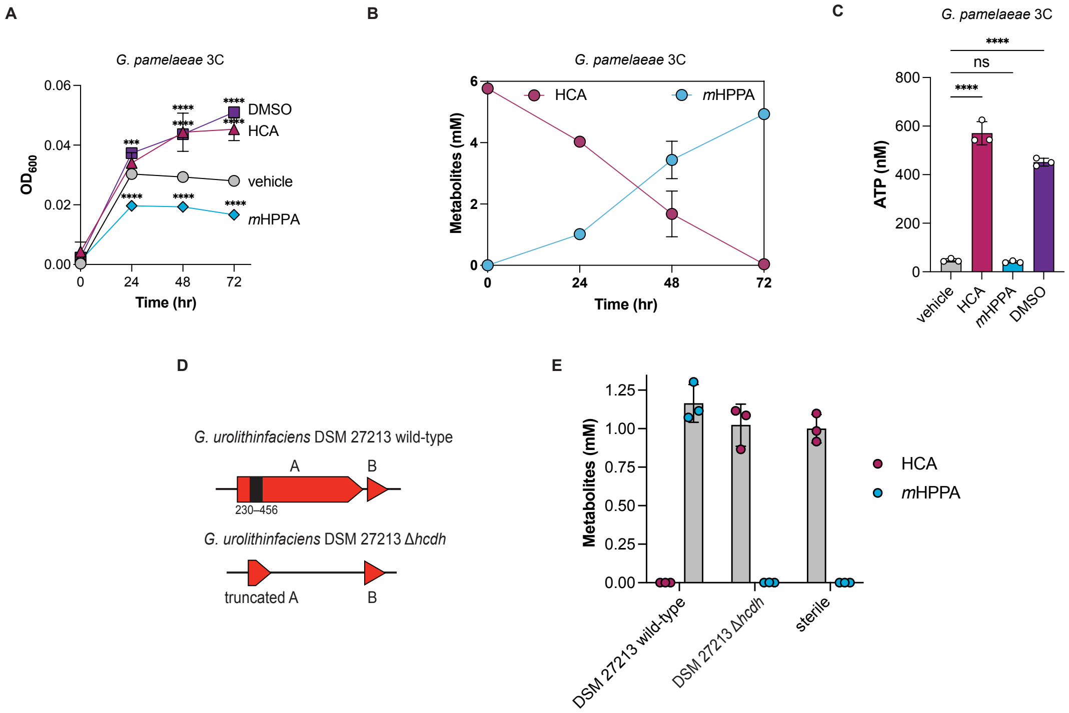

Figure S4. A biological role for *Gp* Hcdh activity in *Gordonibacter* host organisms, related to Figure 3. (A) The effects of HCA, *m*HPPA, and DMSO on the growth of *G. pamelaeae* 3C under anaerobic conditions in 50% BHI medium containing 10 mM sodium formate and additives (vehicle, 5 mM HCA, 5 mM *m*HPPA, or 14 mM DMSO). (B) *G. pamelaeae* 3C metabolism of HCA to *m*HPPA measured by LC–MS/MS (C) ATP production in response to 1 mM HCA, 1 mM *m*HPPA, and 14 mM DMSO in the cell suspension of *G. pamelaeae* 3C grown anaerobically with HCA. (D) A frameshift mutation (Δ230–456) led to the truncation of the catalytic subunit A of Hcdh in *G. urolithinfaciens* DSM 27213. (E) The HCA dehydroxylation activity assay of the *G. urolithinfaciens* wild-type and Δ*hcdh* strains (A–C and E), n=3 biologically independent replicates, data presented are mean ± standard deviation. (A and C) One-way ANOVA followed by Dunnett's multiple comparisons test with all comparisons made against vehicle control. ns, not significant; ***, adjusted p-value < 0.001; ****, adjusted p-value < 0.0001. HCA, hydrocaffeic acid; *m*HPPA, 3-hydroxyphenyl propionic acid; BHI, Brain–Heart Infusion.
